# Design and optimization of a climate-resilient hybrid renewable microgrid for rural electrification in flood-affected regions

**DOI:** 10.1038/s41598-026-56377-w

**Published:** 2026-06-11

**Authors:** Prapti Das, Taib Hasan, Md. Feroz Ali, Obaidullah Obaidi, Md Shafiul Alam, Mohammad Ali, Imil Hamda Imran, Md Kamrul Islam

**Affiliations:** 1 Department of Electrical and Electronic Engineering, Pabna University of Science and Technology, Pabna, 6600 Bangladesh; 2 Department of Energy Engineering, Faculty of Engineering, Kabul University, Kabul, 1006 Afghanistan; 3 Department of Electrical Engineering, College of Engineering, King Faisal University, Al Ahsa, 31982 Saudi Arabia; 4 Department of Civil and Environmental Engineering, College of Engineering, King Faisal University, Al Ahsa, 31982 Saudi Arabia

**Keywords:** Battery energy storage system, Carbon emission mitigation, Grid-connected microgrid, Hybrid renewable energy microgrid, Techno-economic assessment, Energy science and technology, Engineering

## Abstract

Seasonal flooding in the wetland regions of rural Bangladesh frequently disrupts national grid infrastructure, leaving hundreds of thousands of households without reliable electricity access for extended periods, a critical energy vulnerability that conventional grid extension strategies have consistently failed to resolve. To address this challenge, this study designs and optimizes a hybrid renewable energy microgrid combining solar photovoltaic (PV) panels, wind turbines (WT), and a battery energy storage system (BESS), connected to the existing grid via a power converter, to provide reliable and affordable electricity to 200 rural households in Mithamain Upazila, Kishoreganj District, Bangladesh. The system, modeled using HOMER Pro (version 3.14.2), achieves a competitive cost of energy of $0.02995/kWh, which is comparable to conventional rural electricity tariffs. The system’s net present cost is $107,712.60, with an initial investment of $48,107 and an annual operating cost of $895.93. Additionally, the system produces 73.9% of its energy from renewable sources, which reduces carbon dioxide emissions by 43,675 kg every year. Sensitivity analysis highlights the system’s strength in different weather and economic situations, confirming that hybrid microgrids are a feasible and resilient solution for energy access in rural areas that have been hit by floods. This research demonstrates that hybrid renewable energy microgrids are a long-term, low-cost solution for rural electrification in flood-prone areas.

## Introduction

Bangladesh is quite sensitive to climate change since it has high winds, tropical cyclones that happen often, and flooding along its rivers and coasts^1^. These climate dangers make it very hard for energy delivery systems to work, especially in rural and flood-prone areas, where getting reliable electricity is still hard^2^. These rural communities frequently depend on national grid systems, which are regularly disrupted by seasonal flooding, resulting in long-term energy insecurity^3^. With a large amount of power comes from imported fossil fuels, which can be expensive and hard to get, Bangladesh’s energy infrastructure is under strain^4,5^. This significant reliance on imported fossil fuels raises concerns about energy safety and the environment^6^. Renewable energy sources, especially solar and wind power, present viable options for electrifying rural areas^7^. The installed capacity of RE technologies in Bangladesh by 2025, comparing off-grid and on-grid contributions, shows solar leading, followed by hydro and wind, with biomass and biogas having much lower capacities^8^. In 2024, solar energy dominates Bangladesh’s RE mix, contributing 81.90%, followed by wind at 14.10% and hydro at 3.90%^8^. This distribution highlights the growing reliance on solar energy as the country’s main RE resource. However, the intermittent nature of renewable resources requires the integration of BESS to maintain voltage and frequency stability and ensure a continuous electricity supply during resource fluctuations^9^. Given the intermittent nature of these renewable resources and the complex energy demands of flood-prone rural communities, a systematic and data-driven approach to system design is essential, one that can evaluate multiple configurations, load profiles, and resource availability simultaneously to identify the most technically and economically viable solution^10–12^.

A growing body of literature has investigated hybrid renewable energy systems (HRES) across diverse geographic and climatic contexts. International studies have demonstrated the techno-economic viability of HRES in offshore coastal settings^13^, remote villages and islands^14,19,22,24–27,30,41,45,46^, mountainous and inland communities^15–17^, institutional and campus loads^29,32,33,43^, industrial estates and factories^21,39^, urban and grid-connected residential settings^20,35,37,38,40^, and large-scale resource-rich applications^34,36,44^. Within South Asia, several Bangladesh-focused works have been particularly influential. Das et al.^15^ reported a PV/biomass/BESS/WT/hydrokinetic system for a mountainous Bangladeshi region with a COE of $0.128/kWh and 5.31-year payback, Islam et al.^16^ achieved a grid-connected COE of $0.03/kWh and 100% RF for off-grid Bandarban, Ali et al.^18^ obtained a COE of $0.0232/kWh and 80.1% RF for a PV/biomass/WT/grid microgrid in Pabna, Mojumder et al.^23^ benchmarked rural-Bangladesh hybrid options at $0.0647/kWh on-grid, Ali et al.^28^ reported a 100% RF PV/WT/BESS system for Char Kahnpur with NPC of $171,720, Ali et al.^31^ presented a 1.5 MW PV plant for Char Jazira with 671.7% ROI, and Dey et al.^42^ found PV/biomass/BESS to be the most cost-effective off-grid option for Char Amanullah. Most directly relevant are the six Bangladesh-specific HRES studies by Chowdhury and co-workers^47–52^, which include a stand-alone DG/PV/WT/battery HRES for the Kutupalong Rohingya camp^47^ with a COE of $0.35/kWh and 87% RF, a PV/WT/diesel/battery health-care system for Saint Martin’s Island^48^ with a COE of $0.4688/kWh, a resilience analysis of a PV/battery HCC in the Rohingya camp^49^ with 69% emission reduction in resilient mode, a standalone PV/biogas/WT system for Monpura Island^50^ with 99% emission reduction over the existing supply, a solar-PV irrigation pumping study for four Bangladeshi divisions^51^ at 100% RF, and a 5 MW grid-connected solar PV plant for HSIA and SMIA airports^52^ offsetting 3,926 and 3,827.5 tCO₂/yr respectively. Collectively, these studies establish the technical and economic viability of HRES in Bangladesh and globally across refugee, island, irrigation, airport, mountainous, char and inland-rural applications.

Despite these contributions, none of the studies reviewed in^13–52^ addresses the specific energy-access challenge of haor wetland communities, where seasonal monsoon flooding repeatedly disrupts the national grid for households that are nominally grid-connected. The off-grid Bangladesh studies^28,42,47,48,50^ do not model bidirectional grid interaction or grid reliability at all, whereas the grid-connected Bangladesh studies^16,18,23,31,32^ target mountainous, inland or institutional loads and do not treat grid-failure frequency or grid mean repair time as monsoon-driven sensitivity variables. Studies^49,51^ address resilience but for a refugee health-care facility and irrigation-pumping loads respectively, rather than for a residential community load. International HRES studies^13,14,17,19–27,29,30,33–41,43–46^ are similarly situated in coastal-offshore, arid, mountainous, urban, campus, industrial or arctic contexts and therefore do not capture the tropical-wetland flood-resilience requirement. None of the existing works simultaneously combine a grid-connected PV-WT-BESS-converter architecture with site-specific flood-resilience modeling and a multi-parameter sensitivity analysis for a wetland haor village. The present study fills this gap by designing and optimizing a climate-resilient hybrid microgrid for 200 flood-vulnerable rural households in Mithamain Upazila, Kishoreganj — a haor location and load type absent from the existing HRES literature — and achieves a competitive COE of $0.02995/kWh, an NPC of $107,712.60, a 73.9% renewable fraction and an annual CO₂ reduction of 43,675 kg, while explicitly modeling 400 grid outages per year with a one-hour mean repair time and ± 50% variation. Table 1 summarizes the techno-economic and environmental findings of all the reviewed studies alongside the present work and makes the gap addressed by this study explicit for each reference.

**Table 1:** Comparative summary of recently published HRES studies and the present work.

Ref.	Study (Year)	Location	Configuration	COE ($/kWh)	NPC ($)	RF (%)	CO₂ reduction	Limitation / gap vs. present work
^13^	Kangaji et al. (2025)	Buffels Bay, South Africa	Offshore Wind/Tidal/Grid	0.595	$1.74 M	—	Not reported	Coastal offshore HRES; not transferable to inland haor wetland load
^14^	Puppala et al. (2024)	Andhra Pradesh, India	PV/WT/Battery/Converter	0.164	$259,301	—	127,929 kg/yr	Diesel replacement; no flood-outage modeling
^15^	Das et al. (2023)	Mountainous Bangladesh	PV/Biomass/BESS/WT/Hydrokinetic	0.128	$303,306	—	17.6 kg/yr	Mountainous context; no monsoon grid-failure scenario
^16^	Islam et al. (2024)	Bandarban, Bangladesh	PV/Hydro/Battery (off/on-grid)	0.03 (on-grid)	$−199,317	100 (off)	Implicit via RF	Hilly region; no haor-wetland resilience focus
^17^	Zhou et al. (2025)	Inner Mongolia, China	WT/PV/Storage/Grid	0.295 CNY	1.88 M CNY	74.7	68.9%	Arid grassland; not flood-vulnerable wetland
^18^	Ali et al. (2024)	Pabna, Bangladesh	PV/Biomass/WT/Grid	0.0232	$321,798	80.1	78,721 kg/yr	Inland community; flood vulnerability not addressed
^19^	Woldegiyorgis et al. (2025)	Oromia, Ethiopia	WT/PV/Diesel	0.326	$1.42 M	—	49.16%	Village in Ethiopia; no grid-failure sensitivity
^20^	Acosta-Banda et al. (2025)	Tehuantepec, Mexico	PV/Grid	MXN 1.43	—	—	121.6 t/yr	Urban grid-tied PV; no rural flood context
^21^	Tezer (2025)	Balıkesir, Turkey	WT/PV/Biogas	0.0708	$104 M	79.4	78%	Industrial factory load; not residential haor
^22^	Said et al. (2024)	Tumbatu Island, Tanzania	PV/WT/Grid	0.09	$4,003,851	—	Not reported	Island stability focus; no flood-outage sensitivity
^23^	Mojumder et al. (2024)	Rural Bangladesh (generic)	PV/WT/Grid and Off-grid	0.0647 (on-grid)	$1.12 M	100 (off)	Implicit via RF	Generic rural; no haor seasonality modeling
^24^	Rinaldi et al. (2021)	Campo Serio, Peru	PV/WT/Diesel	0.478	$227,335	94	6.1% vs. diesel	Remote Peruvian villages; not flood-vulnerable
^25^	Mukundufite et al. (2024)	Remera, Rwanda	Smart Microgrid	0.186 (subsidy)	—	—	Not reported	Subsidy-dependent dispatch; no grid-failure modeling
^26^	Ribó-Pérez et al. (2021)	Honduras & Zambia	Biomass/PV	0.06 / 0.48	—	—	Not reported	Gasifier modeling focus; off-grid only
^27^	Janke et al. (2025)	Sanirajak, Canada	Wind/Solar/Hydrogen (PGM-H₂)	—	—	97.6 REP	Not reported	Arctic seasonal H₂ storage; not tropical haor
^28^	Ali et al. (2025)	Char Kahnpur, Bangladesh	PV/WT/BESS (off-grid)	0.0688	$171,720	100	Zero emission	Off-grid char; no grid interaction or repair-time sensitivity
^29^	Khaled et al. (2024)	Lahore, Pakistan	PV/BESS/Grid	0.0176	$483,112	99.3	Not reported	University campus; not rural haor residential load
^30^	Chaichan et al. (2025)	Koh Hang, Thailand	PV/WT/Biomass/BESS	0.215	—	100	Avoided	Remote island; no grid-failure scenario
^31^	Ali et al. (2023)	Char Jazira, Bangladesh	1.5 MW PV/Grid	2.82 BDT	—	78.63	37,647 t lifetime	Utility PV plant; no BESS or community-load focus
^32^	Riayatsyah et al. (2022)	Aceh, Indonesia	PV/Grid (campus)	0.0446	$829,692	82	143,452 kg/yr	Campus on-grid; no haor flood vulnerability
^33^	Awan et al. (2024)	Sialkot, Pakistan	PV/Diesel/Grid	—	$116,987	95	Not reported	University campus; no rural community focus
^34^	Aljishi et al. (2025)	Saudi Arabia	WT/PV/BESS	0.128	$842,375	—	Not reported	Arid direct-air-capture sites; not haor wetland
^35^	Ross-Hopley et al. (2024)	Saskatchewan, Canada	HRES/Grid (irrigation)	0.02	—	—	Not reported	Irrigation application; not residential community
^36^	Kiani et al. (2025)	Iran & Switzerland	HRES (multi-site)	Variable	Variable	—	Not reported	Tourism complexes; not rural haor residential
^37^	Chegari et al. (2023)	Residential buildings	PV/WT/PHS/BESS	—	—	—	39% self-sufficiency gain	Single building scale; no community or flood context
^38^	Syed et al. (2025)	Guntur, India	PV/WT/Conventional	—	₹8.15B	—	Not reported	Generator hybrid; no flood-outage modeling
^39^	Shah et al. (2025)	Gujranwala, Pakistan	PV/Diesel/Grid	₹7.29	₹33.90 M	58.8	Not reported	Industrial estate; no rural haor focus
^40^	Ferhan et al. (2025)	On-grid India	PV/WT/Biogas	₹3.56	₹78.64 M	79.7	Not reported	GA-optimized on-grid; no flood-resilience modeling
^41^	Oubouch et al. (2024)	Tazarine, Morocco	PV/WT/PHS	0.03831	$262,596	—	Not reported	Desert rural; no monsoon grid disruption
^42^	Dey et al. (2024)	Char Amanullah, Bangladesh	PV/Biomass/BESS (off-grid)	0.197	$473,582	—	Significant	Off-grid char; no grid-failure sensitivity
^43^	Rhenals-Julio et al. (2025)	Colombia	PV/Hydro/Grid	0.137	—	36.6	Not reported	University campus; no rural flood context
^44^	Panbechi et al. (2025)	Chalus, Iran	Biogas/Solar	0.156 (subsidy)	$718,427	—	Not reported	Subsidy-dependent; no grid-failure modeling
^45^	Vajari et al. (2024)	Repulse Bay, Nunavut, Canada	WT/PV	—	$20.66 M	75	60% GHG	Arctic off-grid; not tropical haor
^46^	Nadeem et al. (2024)	Tharparkar, Pakistan	PV/Biomass (off-grid)	0.104	$95,858	—	Not reported	Off-grid desert; no grid-failure modeling
^47^	Chowdhury et al. (2020)	Kutupalong refugee camp	DG/PV/WT/BESS (off-grid)	0.35	$234,328	87	65/84/61% vs. grid/diesel/kerosene	Off-grid refugee context; no grid interaction or flood-outage modeling
^48^	Chowdhury et al. (2021)	Saint Martin’s Island	PV/WT/Diesel/BESS (off-grid)	0.4688	$69.38 M	28	27% vs. diesel	Isolated island HCC; no monsoon grid-failure scenario
^49^	Chowdhury et al. (2023)	Rohingya refugee camp HCC	PV/Battery	—	—	—	69% (resilient) / 44% (financial)	Refugee HCC resilience, not haor residential community
^50^	Chowdhury et al. (2022)	Monpura Island, Bhola	PV/Biogas/WT (off-grid)	0.691	$141 M	—	99% vs. existing	Off-grid coastal island; no grid-failure sensitivity
^51^	H. Chowdhury et al. (2022)	Rajshahi/Sylhet/Dhaka/Chattogram	Solar-PV irrigation	0.1496–0.1576	—	100	Not reported	Irrigation pumping; not residential haor load
^52^	Chowdhury et al. (2021)	HSIA & SMIA airports	5 MW grid-tied PV	—	—	—	3,926 & 3,827.5 t/yr	Airport scale; no BESS, no rural/wetland context
This work	Present study (2026)	Mithamain Upazila, Kishoreganj (haor)	PV/WT/BESS/Converter/Grid	0.02995	$107,712.60	73.9	43,675 kg/yr	Climate-resilient grid-tied microgrid for 200 flood-vulnerable households with explicit grid-failure-frequency and repair-time sensitivity

While a growing body of research has investigated HRES configurations for rural electrification in Bangladesh, a critical and underexplored gap remains in the literature. Studies such as Chowdhury et al.^47–50^, focus predominantly on off-grid island or refugee camp contexts, which, despite facing energy access challenges, do not experience the specific hydrological and infrastructural disruptions caused by seasonal monsoon flooding in haor wetland areas. These studies neither model grid-connected systems nor simulate flood-induced grid outage patterns, two defining features of the energy challenge in regions such as Mithamain Upazila, Kishoreganj. Similarly, while Hemal Chowdhury et al.^51^ incorporate environmental impact assessment in the Bangladesh rural electrification context, they do not address the dynamic interaction between flood seasonality and grid reliability, nor do they evaluate BESS performance under monsoon-driven generation variability. Studies on PV systems for institutional or airport applications in Bangladesh^52^ are further removed from the community-scale residential load profiles and flood-resilience requirements of the haor communities studied here. Moreover, none of the existing Bangladesh-focused HRES studies simultaneously address: (i) a grid-connected PV-WT-BESS microgrid architecture specifically designed to maintain a continuous supply during flood-induced grid outages; (ii) a site-specific sensitivity analysis that explicitly includes grid failure frequency and repair time as variable parameters reflecting monsoon seasonality; and (iii) a multi-parameter techno-economic and environmental optimization for a wetland haor community load profile in Kishoreganj District. The present study directly addresses these gaps by designing and optimizing a climate-resilient hybrid microgrid for 200 flood-vulnerable rural households in Mithamain Upazila, a location type entirely absents from the existing HRES literature for Bangladesh, with explicit modeling of seasonal grid disruptions, a comprehensive 11-parameter sensitivity analysis, and a rigorous techno-economic evaluation that achieves a competitive COE of $0.02995/kWh and a renewable fraction of 73.9%.

This study aims to fill these gaps by designing a hybrid RE microgrid for 200 households in Mithamain Upazila, a flood-prone area in Bangladesh. The key objectives of the research are: first, to develop and optimize a HRES combining PV, WT, and BESS to ensure a stable power supply during flooding-induced grid outages. Second, the study performs a thorough techno-economic analysis, assessing the cost efficiency by calculating the NPC and COE. Finally, the environmental impact is evaluated, particularly regarding emissions reduction, in alignment with national and global sustainable development targets. Additionally, the study includes a sensitivity analysis to evaluate how variables like solar radiation, availability of wind, and system costs affect the system’s performance. The findings of this study aim to demonstrate the viability of hybrid microgrids as a long-term sustainable approach for providing electricity to rural areas in Bangladesh, with potential for application in other flood-prone regions globally.

## Materials and methodology

### Site location

**Fig. 1:**
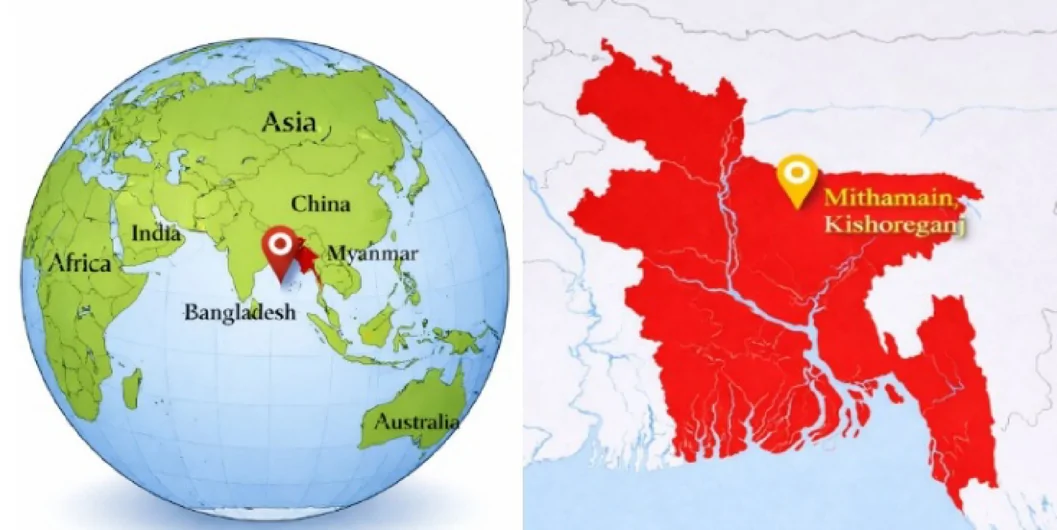
Geographic location of the study area.

The case analysis is conducted in Mithamain Upazila, Kishoreganj District, Bangladesh (24°25.3′N, 91°2.3′E), focusing on the load profile of 200 rural households. The community has a daily average demand of 440.88 kWh and a peak load of 60.42 kW. Situated in a wetland (haor) area with plentiful solar and wind energy resources but inconsistent grid access, this location is ideal for evaluating sustainable hybrid renewable microgrid solutions. The site’s unique conditions, as depicted in Fig. 1, make it a suitable candidate for testing such energy systems. The geographical map was prepared using the study-area coordinates of Mithamain Upazila, Kishoreganj District, Bangladesh, obtained from Google Maps/Google Earth and created using Draw.io software. The location was selected because Mithamain is part of the Haor wetland region, where seasonal flooding frequently affects grid accessibility and electricity reliability.

### Energy consumption profile

**Fig. 2:**
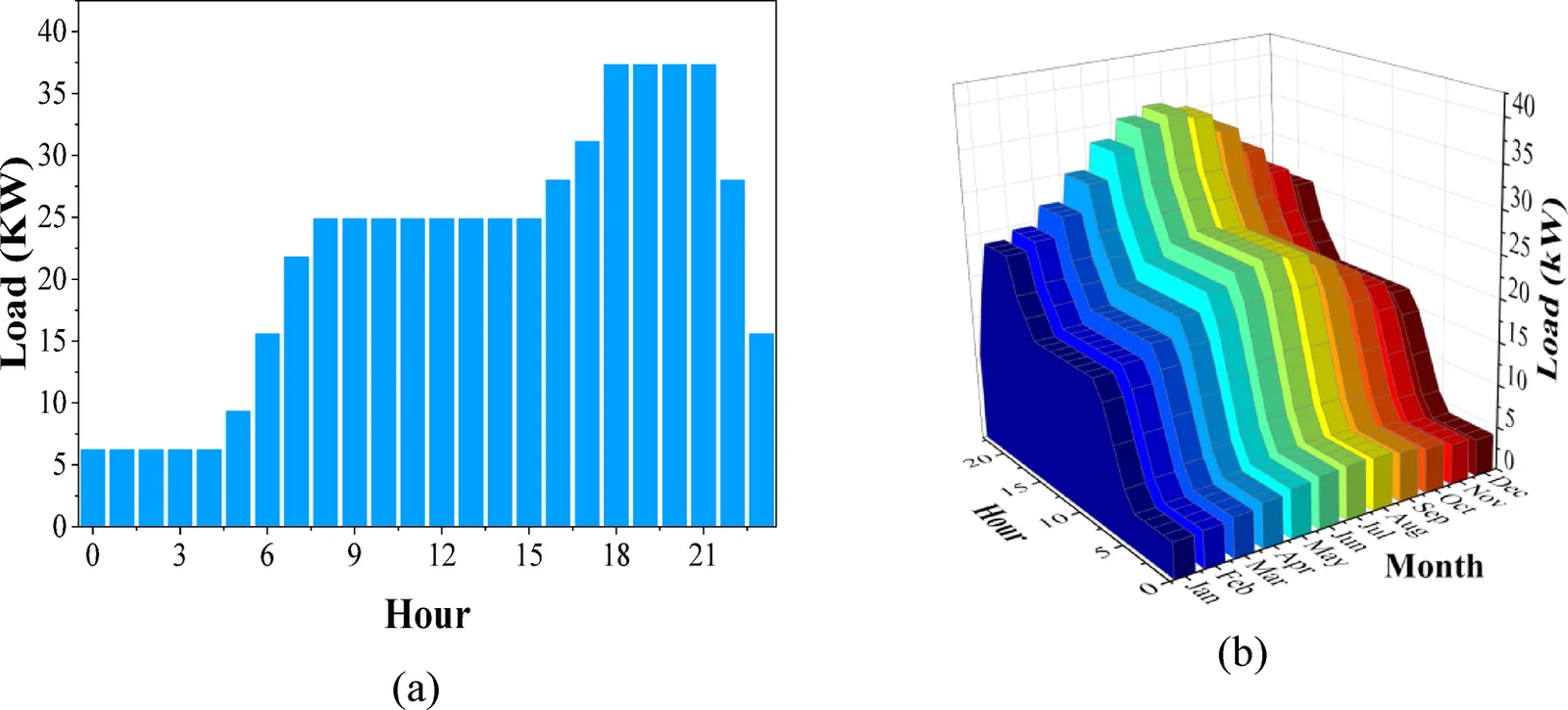
Load profile for community Load: (**a**) Daily (**b**) Monthly.

The load profile in Table 31 represents the typical electric power usage pattern of rural households. It includes essential appliances such as lights, fans, phone chargers, televisions, and shared refrigerators. Based on the number of units, operating hours, and power ratings across 200 homes, the total daily energy consumption was estimated at 440.88 kWh. Fans and lighting account for the majority of the load due to their frequent use and longer operating durations. This load assessment provides a realistic estimation of rural household electricity demand, serving as a baseline for the design and performance optimization of a reliable and sustainable HRES. The household load data presented in Table 2; Fig. 2 were collected through a structured field survey of 200 rural households in Mithamain Upazila, employing the bottom-up load estimation methodology in which total energy demand is computed by aggregating the rated power and daily usage hours of all identified household appliances^15,53^.

**Table 2:** Estimated load profile of 200 rural households.

Electrical appliances	Quantity per house	Operating hours per day	No. of houses	Power ratings (w)	Total units	Consumption (kWh/d)
Light	4	6	200	15	800	72
Fan	2	9	200	60	400	216
Phone Charger	3	3	200	5.2	600	9.36
Tv	1	3	150	80	150	36
Freeze (shared)	1	24	40	112	40	107.52
Total						= 440.88

Figure 2 illustrates the community load profile in daily and monthly formats. Figure 2a displays hourly load variations, with peaks in the afternoon and evening, and Fig. 2b illustrates seasonal demand fluctuations, providing insights for optimizing energy systems. These profiles optimize peak demand and distribution.

To model RE systems in HOMER, reliable data on solar radiation, wind speed and temperature data are required. Monthly solar radiation averages, clearness index database and daily radiation data were taken from NASA’s POWER (Prediction of Worldwide Energy Resource) database for the project location^54^. This data covers a 22-year period (1983–2005) and is specific to the location’s coordinates (latitude: 24.25, longitude: 91.25). Additionally, wind speed measurements taken at 50 m above ground level, was sourced from the same database, spanning 30 years (1984–2013). The temperature data, representing monthly averages of daily temperatures from January 1984 to December 2013, was also extracted. HOMER Pro software takes long-term averaged climate parameters as input data and can simulate the performance of renewable energy-based systems, providing an accurate representation of how the system will perform based on site-specific weather information and available energy resources.

#### Solar irradiance and monthly clearness index

**Fig. 3:**
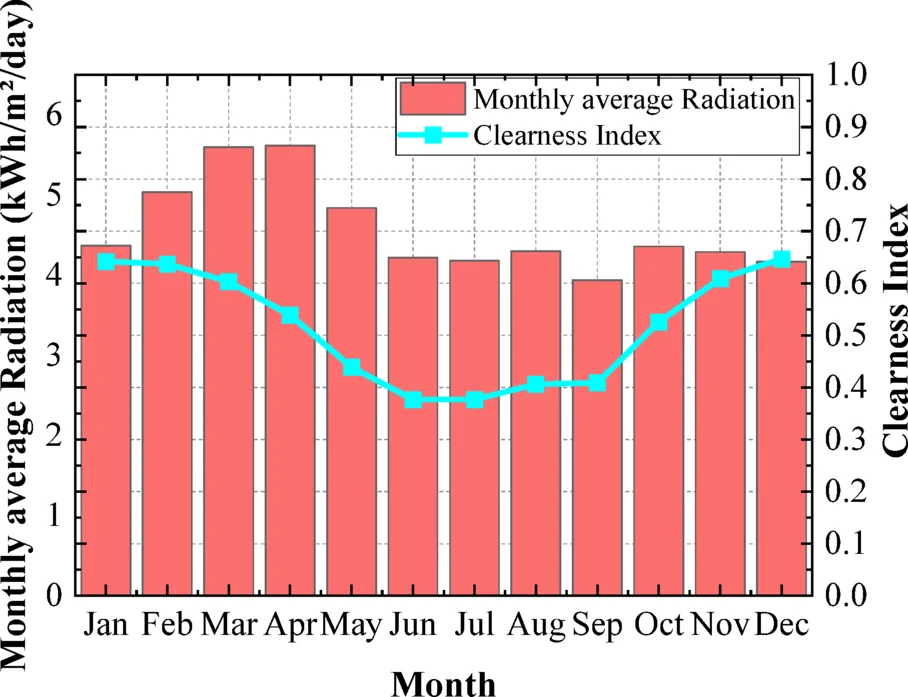
Site-specific solar GHI and clearness index.

The Solar GHI data, shown in the graph of Fig. 3 represents the average daily radiation per month in kWh/m²/day and the clearness index. This data, collected from NASA’s POWER database^55^, indicates that the highest solar radiation occurs during the months of February to April, while the minimum occurs in June and July, and the site experiences an annual average solar irradiation of 4.58 kWh/m²/day. The yellow curve shows the clearness index, which measures the fraction of sunlight reaching the Earth’s surface. The clearness index peaks during the months of November and December, reflecting clearer skies and higher solar radiation availability in these months. The efficiency of PV systems is closely tied to these factors. While higher solar radiation enhances energy generation potential, the clearness index influences the system’s efficiency by the amount of solar energy hitting the panels due to atmospheric variations.

#### Wind speed

**Fig. 4:**
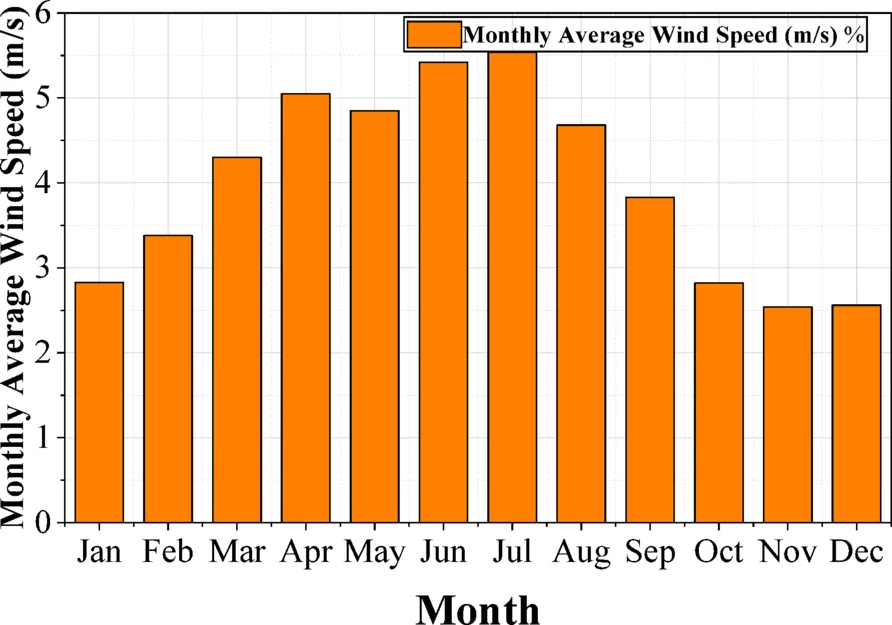
Monthly wind speed averages for the site.

The monthly variation of average wind speed for the selected site is presented in Fig. 4. Monthly average wind speed at the study site (Mithamain Upazila, Kishoreganj) derived from the NASA POWER database^55^ at coordinates 24.20°N, 90.91°E. The wind speed is highest in the summer period of June and July, reaching up to 5.5 m/s, while the lowest values are observed in the winter months, particularly from November to December, with speeds dropping to around 2.5 m/s. The annual average wind speed is 3.98 m/s. These fluctuations are important for wind energy systems, as higher wind speeds during summer months provide greater energy generation potential, while the lowest values recorded in the winter season may reduce energy output from wind turbines during that period.

#### Temperature

**Fig. 5:**
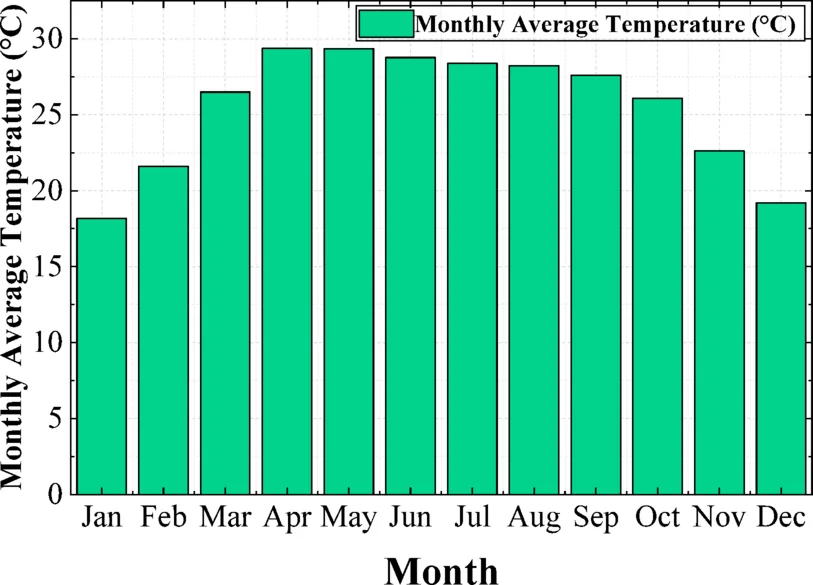
Monthly average temperature for the site.

Figure 5 illustrates the monthly variation of the daily average temperature at the selected location. The temperature data were collected from NASA’s POWER database^55^ for the study-area coordinates and represent long-term monthly average values. The highest temperatures occur between March and May, reaching values around 29 °C, while the lowest temperatures are observed in January, averaging just over 18 °C. The annual average monthly temperature for the site is 25.49 °C. These temperature variations are significant for energy system simulations, as temperature affects both the operational efficiency of energy-producing systems, such as PV, and the energy demand throughout the year. Higher temperatures can lead to increased cooling energy demand, while lower temperatures might require additional heating.

### HOMER Pro

**Fig. 6:**
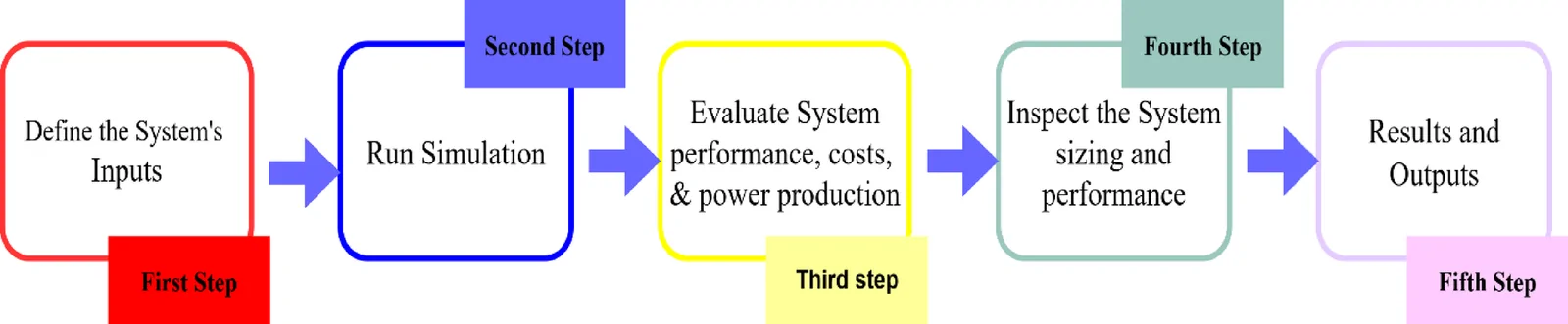
HOMER Pro Software Architecture.

HOMER Pro (Hybrid Optimization of Multiple Energy Resources) is a widely recognized software solution for the design and performance optimization of microgrids, integrating RE sources, BESS, and conventional generators^56^. It was developed by the National Renewable Energy Laboratory (NREL) in the United States^57^. It implements three primary functions: modeling, optimization, and sensitivity assessment, and allows users to model various power configurations, including PV, WT, BESS, and DG^58^. HOMER Pro is widely employed for conducting techno-economic analysis of HRES, evaluating metrics such as NPC, COE, RF, and O&M^59^. The software supports both isolated and grid-integrated system configurations, facilitating the assessment of dispatch strategies, energy flows, and reliability constraints^60^. Through its simulation, optimization, and sensitivity analysis capabilities, HOMER Pro aids in identifying cost-effective system architectures under varying scenarios^61^. Figure 6 illustrates a simplified flowchart representing the optimization process of HOMER Pro^62^.

The economic performance of the proposed HRES is evaluated using the NPC and the LCOE, also referred to as the COE. The NPC represents the total lifetime cost of the system, including the initial capital investment and the discounted annual operation, system maintenance, and fuel costs associated with the project lifespan T at a discount rate r. The COE provides the average electricity generation cost per unit, calculated by dividing the total lifetime expenditures by the overall electricity production. Equations (1) and (2)^63,64^ represent the model formulations for calculating NPC and COE in HOMER pro, respectively.

1 $$\:NPC={C}_{\mathrm{cap\:}}+\sum\:_{t=1}^{T}\:\frac{{C}_{om}\left(t\right)+{C}_{\mathrm{fuel\:}}\left(t\right)}{(1+r{)}^{t}}$$

2 $$\:COE=\frac{\sum\:_{t=1}^{T}\:\left({C}_{cap}+{C}_{om}\left(t\right)+{C}_{\mathrm{fuel\:}}\left(t\right)\right)}{\sum\:_{t=1}^{T}\:{E}_{\mathrm{total\:}}\left(t\right)}$$

Where T denotes the duration of the project in years, reflecting the period over which costs and energy production are evaluated. $\:{C}_{cap\:}$is the upfront capital investment required for the procurement and installation of the energy system components. $\:{C}_{om}\left(t\right)$ corresponds to the annual operation and maintenance costs incurred in year t, accounting for routine servicing, repairs, and other recurring expenditures necessary to ensure reliable system performance. $\:{C}_{fuel}\left(t\right)\:\:$is the fuel-related expenses for the same year, which are particularly relevant for hybrid systems incorporating conventional generators. * r* is the discount rate applied to reflect the time-based value of money, reflecting the present value of future costs. $\:{E}_{total}\left(t\right)$ denotes the total electricity produced by the system in year t, measured in kWh, serving as the basis for assessing cost-effectiveness and levelized energy metrics over the system’s lifetime.

**Fig. 7:**
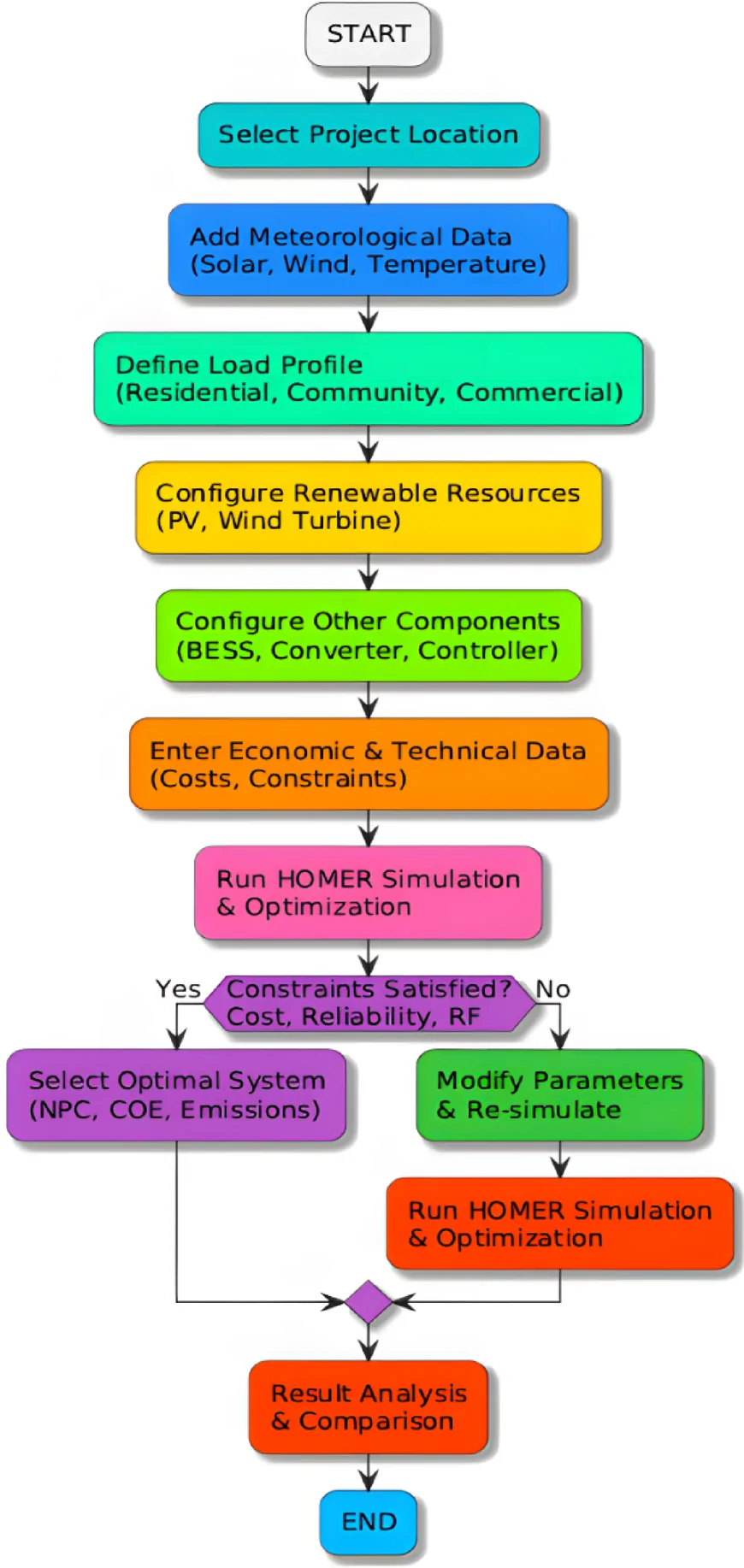
Methodology flowchart for the proposed work.

Figure 7 outlines the flowchart of the steps involved in designing a HRES using simulation software. It starts with selecting the project location and adding relevant meteorological data like solar radiation, wind speed, and temperature. Next, the load profile is defined, considering residential, community, and commercial demand patterns. The system is then configured in two parts: renewable resources and other components such as batteries, converters, and controllers. Economic and technical inputs, including costs, operation, and maintenance, and system constraints, are entered before running the simulation. The software evaluates whether the system meets the required cost, reliability, and renewable fraction. If not, parameters are adjusted, and the simulation is repeated. Once all constraints are satisfied, the optimal system is selected, and results are analyzed for performance, cost-effectiveness, and environmental impact. This structured approach ensures a reliable and practical pathway for hybrid energy planning.

**Fig. 8:**
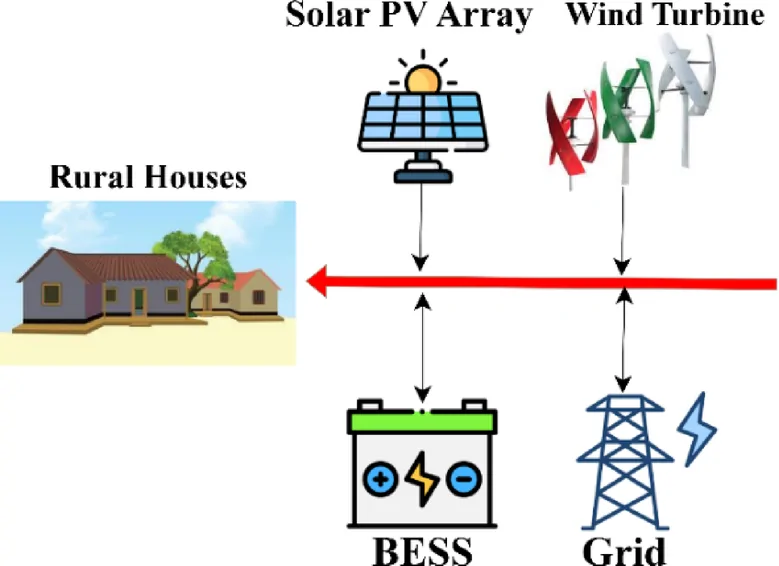
Proposed HRES-based microgrid architecture.

The objective of HRES is to maintain an uninterrupted energy supply with enhanced efficiency by integrating a variety of RE sources, such as WT, PV, generator, BESS, grid power, etc.^65^. Figure 8 illustrates a HRES designed for rural households that combines solar PV arrays and vertical axis WT as RE sources. The system includes a BESS, which stores excess energy to ensure continuous power availability during low generation periods. The electricity generated or stored can be used to meet household energy demands. Furthermore, the system supports bidirectional energy flow, allowing excess power to be fed into the main grid or drawn from it when needed. This architecture ensures a reliable and sustainable energy supply that makes energy access more secure in rural communities.

**Fig. 9:**
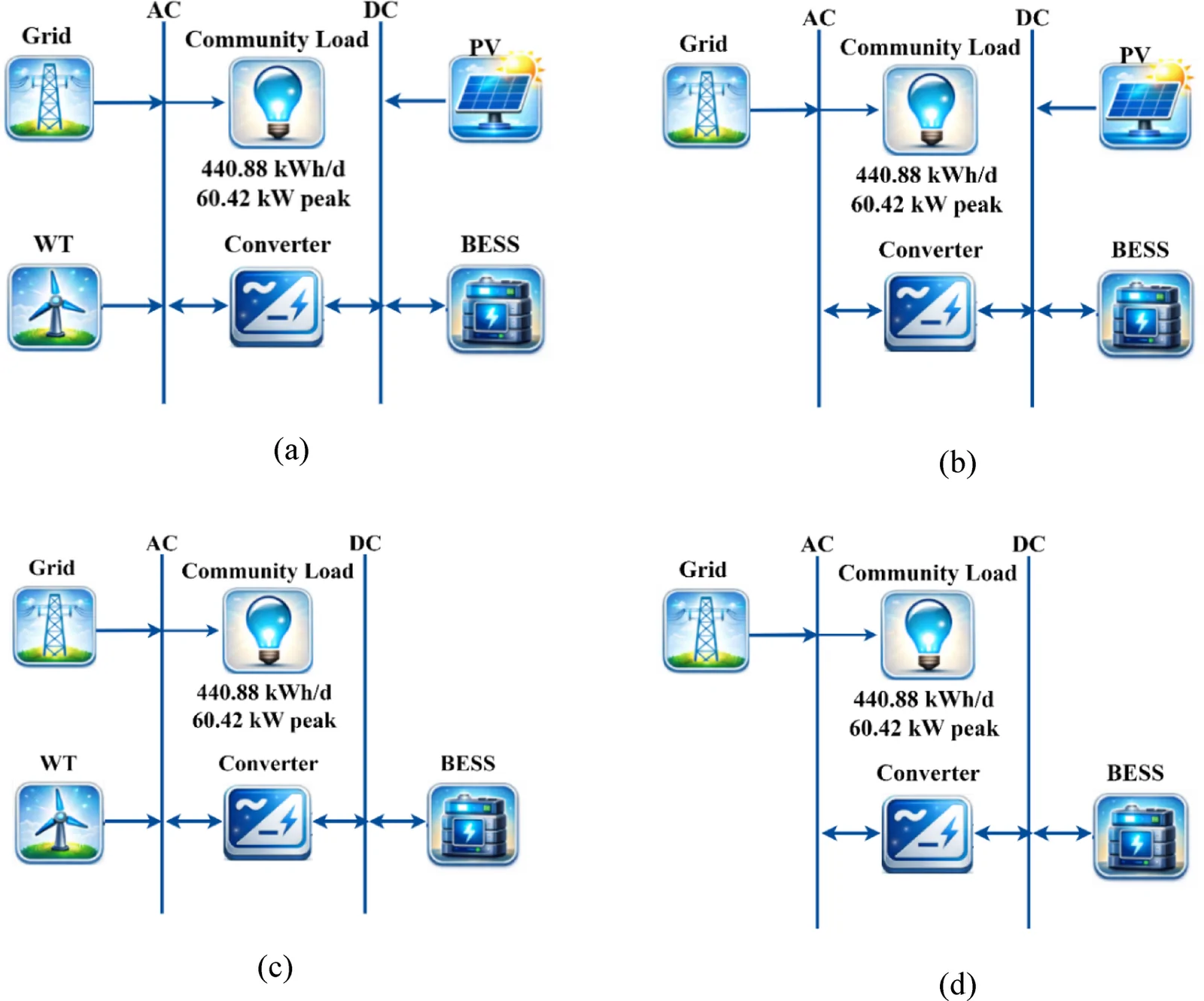
Schematic illustration of various configurations: (**a**) Case-A, (**b**) Case-B, (**c**) Case-C, and (**d**) Case-D.

Figure 9 presents four different microgrid configurations designed to meet the energy needs of a community. In all cases, the AC grid serves as the primary source, supplying a daily load of 440.88 kWh with a peak demand of 60.42 kW. To improve reliability and incorporate RE, a PV system, a WT, and a BESS are connected through a DC bus and linked to the AC grid via a power converter. In Fig. 9a, both PV and WT are integrated with BESS, allowing the system to utilize solar and wind resources simultaneously to meet the load efficiently. Figure 9b includes only PV with BESS, while Fig. 9c relies solely on WT with BESS, demonstrating how individual renewable sources can support the community when combined with storage. Figure 9d excludes renewable generation entirely, depending on the BESS with support from the AC grid. The schematics clearly show the direction of power flow and highlight the essential role of the converter in bridging AC and DC components. Overall, these configurations provide insight into how different combinations of renewable generation and storage can be managed effectively to supply the community load. They demonstrate practical strategies for enhancing energy reliability, reducing dependence on conventional grid sources, and integrating RE in a flexible and efficient way.

### Solar PV

The PV array output power is written in Eq. (3) as^66^:

3 $$\:{P}_{PV}={Y}_{PV}\:\times\:\:{f}_{PV}\:\times\:\left(\frac{{G}_{T}}{{G}_{T,STC}}\right)\times\:\left[1+\:{\alpha\:}_{P}\left({T}_{c}-\:{T}_{C,STC}\right)\right]$$

Here, $\:{Y}_{PV}$ indicates the nominal capacity of the PV system under STC, and $\:{f}_{PV}$ indicate the power reduction factor accounting for system losses caused by dust, electrical wiring, and inverter inefficiencies. $\:{G}_{T}$ and $\:{G}_{T,\:STC}$ correspond to the actual and reference solar irradiance, respectively. $\:{\alpha\:}_{p}$ is the temperature coefficient associated with power output, while $\:{T}_{C}$ and $\:{T}_{C,\:\:STC}$ are the operating PV cell and reference temperatures, influencing output due to thermal variations.

### Wind turbine

The mechanical energy produced by the WT is written in Eq. (4) as^67^:

4 $$\:{P}_{WT}=\frac{1}{2}\rho\:A{C}_{P}\left(\lambda\:,\beta\:\right){v}_{\omega\:}^{3}$$

where *ρ* is the density of air (kg/m³), *A* is the area swept by the rotor blades (m²), $\:{C}_{P}(\lambda\:,\beta\:)$ is the power coefficient dependent on the tip-speed ratio λ and blade pitch angle β, and $\:{v}_{\omega\:}$ is the wind speed (m/s). This equation indicates that the extractable wind power depends on both wind parameters and turbine design features, reflecting the aerodynamic efficiency of transforming wind kinetic energy into mechanical power.

The tip ratio can be expressed by Eq. (5) as^67^:

5 $$\:\lambda\:=\frac{{\omega\:}_{m}R}{{v}_{\omega\:}}$$

Where, $\:{{\upomega\:}}_{\mathrm{m}}$ denotes the angular velocity of the rotor components (rad/s), R is rotor radius (m), and $\:{\mathrm{v}}_{{\upomega\:}}$ is the wind speed.

Additionally, the modeling of wind energy can be expressed using Eq. (6) as^68^:

6 $$\:{E}_{w}=\:\left\{\begin{array}{c}{P}_{rat\:}\left(\frac{{V}_{Z,t}-{V}_{cut-in}}{{V}_{rat}-{V}_{cut-in}}\right)\:\:{V}_{cut-in}<\:{V}_{Z,t}<\:{V}_{cut-off}\\\:0\:\:\:\:\:\:\:\:\:\:\:\:\:\:\:\:\:\:\:\:\:\:\:\:\:{\:\:V}_{Z,t}<\:\:{V}_{cut-in}OR{V}_{Z,t}\ge\:\:{V}_{cut-off}\end{array}\right.$$

Where $\:{\mathrm{P}}_{\mathrm{r}\mathrm{a}\mathrm{t}\:}$ represents the rated power of the WT corresponding to the rated wind speed. $\:{\mathrm{V}}_{\mathrm{Z},\mathrm{t}}$ denotes the wind speed at the hub height ($\:{\mathrm{h}}_{\mathrm{z}}$) of the turbine, which is calculated using Eq. (7), and $\:{\mathrm{V}}_{\mathrm{r}\mathrm{a}\mathrm{t}}$, $\:{\mathrm{V}}_{\mathrm{c}\mathrm{u}\mathrm{t}-\mathrm{i}\mathrm{n}}$, and $\:{\mathrm{V}}_{\mathrm{c}\mathrm{u}\mathrm{t}-\mathrm{o}\mathrm{f}\mathrm{f}}$ correspond to the cut-in and cut-off wind speeds, respectively.

7 $$\:{V}_{Z,t}=\:{V}_{0,t}\:(\:\frac{{h}_{z}}{{h}_{0}}{)\:}^{\alpha\:}$$

Where $\:{\mathrm{V}}_{0,\mathrm{t}}$ is the wind speed at a particular height $\:{\mathrm{h}}_{0}$, and α signifies the wind shear exponent.

### Converter

In microgrid systems, power converters enable the integration of RE sources with BESS units, ensuring effective energy management and optimized power distribution^69^. The output power of a power electronic converter is determined by Eq. (8) as^15^:

8 $$\:{P}_{out}={\eta\:}_{con}{P}_{in}$$

Here, $\:{P}_{out}$ represents the delivered power to the load or DC/AC bus, $\:{P}_{in}$ is the power supplied from the renewable source or upstream system, and $\:{\eta\:}_{con}$ is the converter efficiency, typically expressed as a fraction less than one. This simple relation highlights the inherent losses in conversion due to factors such as switching, conduction, and thermal effects.

### BESS

Surplus energy generated by renewable sources is stored in the battery bank to supply energy during peak demand periods^70^. SOC of a battery represents the available energy in the battery relative to its full capacity, serving as a key indicator for energy management in hybrid microgrids^71^. The formula of SOC can be represented by Eq. (9) as^65^:

9 $$\:SOC\left(t\right)\:=\:SOC(t\:-1)\:\times\:{\int\:}_{t-1}^{t}\frac{{\eta\:\:}_{bat}\times\:\:{L}_{b}\left(t\right)\:}{{V}_{bus}}dt$$

This equation updates SOC based on the previous time step SOC(t − 1), considering the charging or discharging power $\:{\mathrm{L}}_{\mathrm{b}}\left(\mathrm{t}\right)$, battery efficiency $\:{{\upeta\:}\:}_{\mathrm{b}\mathrm{a}\mathrm{t}}\:$and the system voltage $\:{\mathrm{V}}_{\mathrm{b}\mathrm{u}\mathrm{s}}$. The integral over the time interval [t-1, t] captures the net energy exchanged during that period.

### Power supply grid

**Fig. 10:**
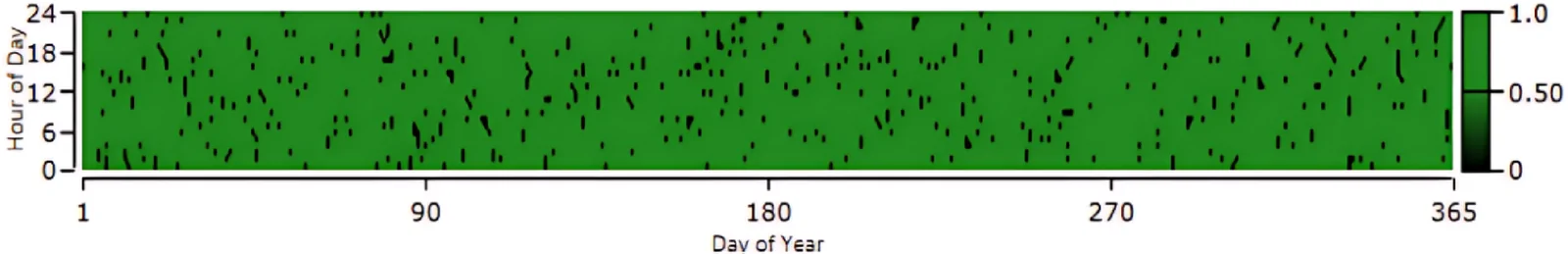
Grid failure scenario in the study location.

According to the Bangladesh Power Supply Regulatory Commission, the electricity buying cost from the grid was fixed at $0.08/kWh, while the sell-back or export rate was set at $0.04/kWh, values that were applied in the HOMER Pro simulation^64^. The grid reliability model accounted for frequent outages, with the mean outage frequency set at 400 times per year. The average repair time was set to 1 h, with a fluctuation of 50%, to reflect the unpredictable character of grid recovery. This was done to represent the irregular availability of the grid in that area. The patterns of interruptions, both scheduled and unscheduled, were visualized to assess the effects of grid reliability on the operation of the energy system. Figure 10 shows the modeled grid failure scenario for the chosen site, showcasing its seasonal fluctuations.

#### Grid emission factor

Based on the reference document issued by the Department of Environment, Ministry of Environment and Forests, Government of Bangladesh (2025), the official Grid Emission Factor (GEF) for Bangladesh is reported as 0.61 tCO₂/MWh for 2019–20 and 0.62 tCO₂/MWh for 2020–21 and 2021–22^72^. In this study, the GEF in HOMER Pro was set to approximately 620 gCO₂/kWh (equivalent to 0.62 tCO₂/MWh), aligning with the national benchmark to provide an accurate measure of the grid electricity’s carbon intensity. This factor also accounts for associated emissions of SO₂ (2.74 g/kWh) and NOₓ (1.34 g/kWh), thereby reflecting the environmental implications of grid-based power consumption in Bangladesh.

### Technical specifications

The key specifications of the PV, WT, converter, and BESS that are essential for evaluating the system’s functional performance in the HOMER Pro simulation are presented in Table 3.

**Table 3:** Components technical specifications.

Input factors	PV	Converter	WT	BESS
Nominal capacity	2 kW	-	1 kW	500 Ah
Efficiency	19%	95%	-	72%
Hub Height	-	-	17 m	
Life Time	25yr	15yr	20yr	30yr

The unit per expenses of the PV, WT, converter, and BESS components, encompassing capital expenditures, replacement costs, and O&M, are referenced from recent sources. These values are used to evaluate the economic feasibility of the microgrid components and are summarized in are presented in Table 4.

**Table 4:** Per unit pricing of equipment.

Parameters	PV	WT	Bess	Converter
Capital cost	$385/kw	$350/kw	$750/kw	$290/kw
Replacement cost	$385/kw	$350/kw	$700/kw	$290/kw
O & M cost	$8/yr	$1/yr	$0/yr	$3/yr
Reference	^73^	^74^	^28^	^18^

## Results and discussion

This simulation analysis explored 2,934 possible microgrid setups. Among them, 748 configurations were considered viable. The most restrictive factor was the constraint on capacity shortfall, which caused 2,186 configurations to be considered infeasible. Furthermore, 652 configurations were excluded from consideration: 429 due to the unavailability of a power conversion device and 207 due to the incorporation of unnecessary converter units. These exclusions underscore the significance of proper component choice and capacity planning for the development of an optimized and reliable microgrid. The study considered a 25-year planning period, utilizing hourly time-series simulations to evaluate a range of possible microgrid scenarios. Four different configurations, integrating PV, WT, BESS, and grid connections, were analyzed to determine the optimal microgrid design strategy in terms of cost-effectiveness. Table 5 summarizes the various configurations, including combinations of PV, WT, BESS, grid, and converters.

**Table 5:** Synthesis of different cases.

Components	Configuration
PV-WT-BESS-Converter-Grid	Case-A
PV-BESS-Converter-Grid	Case-B
WT-BESS-Converter-Grid	Case-C
BESS-Converter-Grid	Base Case

### Techno-economic analysis

**Fig. 11:**
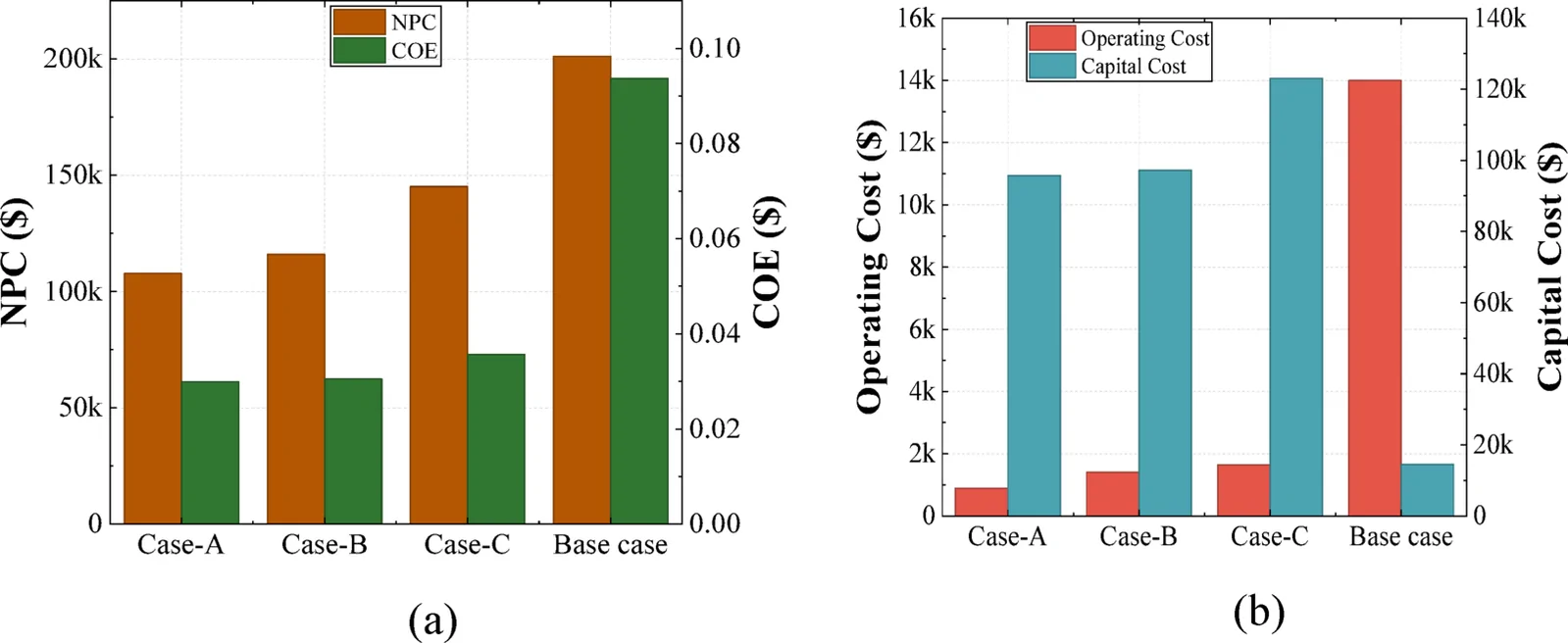
Comparison of key cost factors across different cases: (**a**) NPC and COE, (**b**) Operating and Capital costs.

Figure 11 presents a comparison of four scenarios based on key economic indicators: NPC, COE, annual O&M, and initial capital cost. Figure 11a compares the NPC and COE. The brown bars represent the NPC, which represents the total financial cost over the system’s lifetime, while the green bars represent the COE, indicating the cost per unit of electricity generated. Scenario-A demonstrates the lowest NPC and COE, highlighting its superior cost-effectiveness over the system’s life cycle. Figure 11b compares the initial capital and operating costs. Scenario-A exhibits a balanced economic profile with moderate capital costs and low operating expenses. In contrast, Scenario-B shows higher NPC and COE, with increased operating costs but reduced capital investment. Scenario-C experiences further increases in both NPC and COE, coupled with higher operational costs, while capital investment requirements are lower. Lastly, Scenario-D, despite having the highest operating costs and COE, requires the lowest capital investment. These results suggest that Scenario-A is the most cost-effective, offering an optimal balance between long-term financial sustainability and upfront investment.

**Fig. 12:**
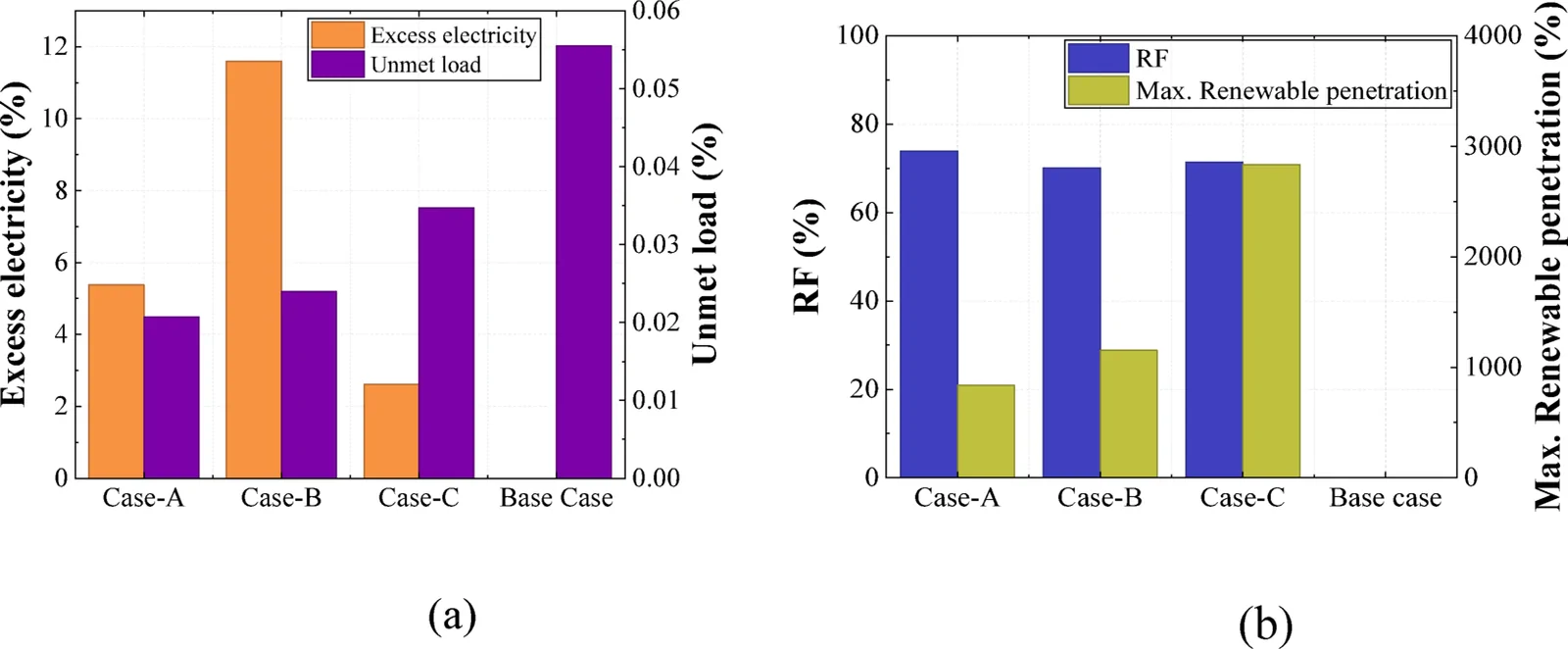
Comparison of different factors across different cases: (a) Excess electricity and Unmet load, (b)Renewable fraction and Renewable penetration.

Figure 12 compares various factors across different cases: (a) Excess electricity and Unmet load, and (b) RF and Max. REP. In Fig. 12a Excess electricity is represented by the orange bars and unmet load by the purple bars. Case-A shows the lowest values for both excess electricity and unmet load, suggesting it operates most efficiently. Case-B exhibits higher levels of excess electricity and unmet load, indicating challenges in balancing energy generation and consumption. Case-C experiences the highest unmet load, implying difficulty in meeting demand, while the base case exhibits the highest values for both excess electricity and unmet load, reflecting poor system performance. In Fig. 12b, the blue bars represent RF, and the yellow bars show maximum renewable penetration. Case-A and Case-B display higher RF but lower penetration, while Case-C achieves the highest REP, though with a slightly lower RF compared to the others.

**Fig. 13:**
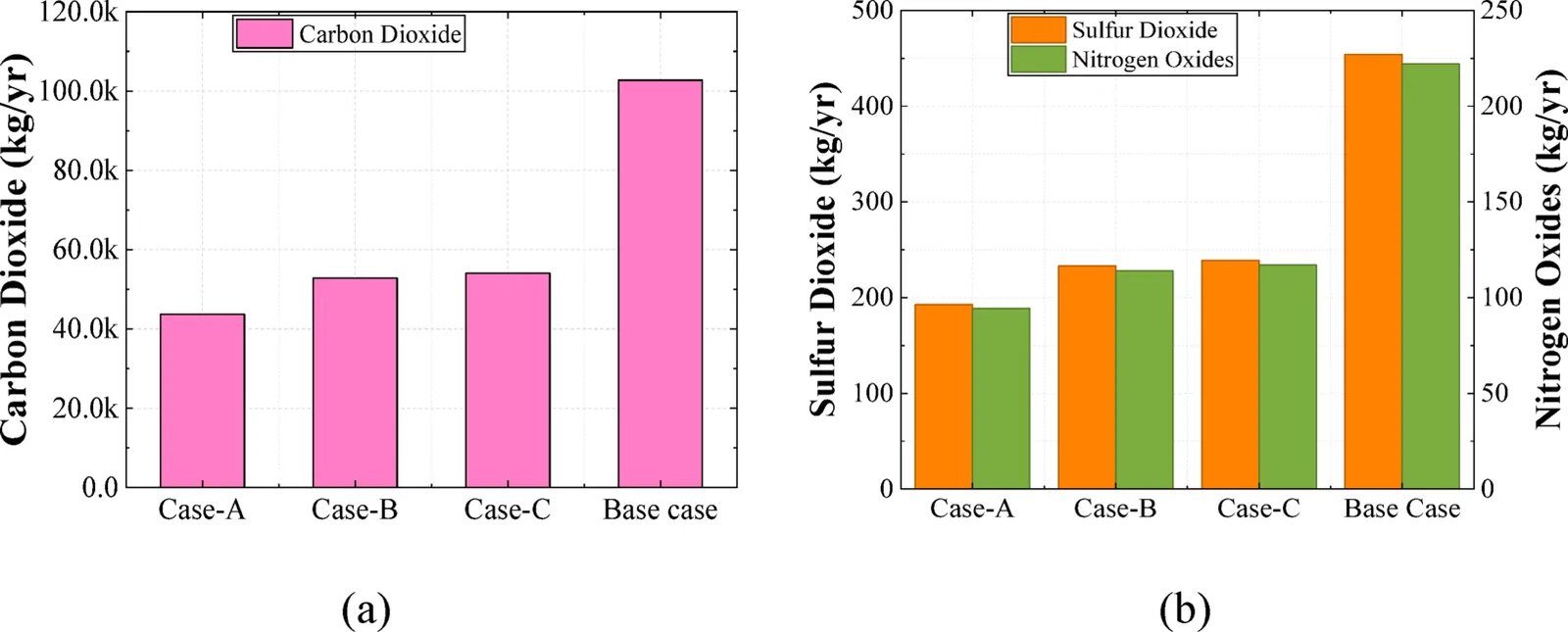
Comparative Analysis of Emissions across different cases: (**a**) Carbon dioxide, (**b**) Sulfur dioxide, and Nitrogen oxides.

Figure 13 compares emissions across different cases: (a) Carbon dioxide and (b) Sulfur dioxide and Nitrogen oxides. In Fig. 13a carbon dioxide emissions are highest for the base case, followed by Case-C, Case-B, and Case-A, which exhibits the lowest emissions, indicating superior environmental performance. Figure 13b compares sulfur dioxide (orange bars) and nitrogen oxides (green bars) emissions. The base case also shows the highest emissions for both pollutants, while case A again demonstrates the lowest sulfur dioxide and nitrogen oxides emissions, suggesting the most environmentally sustainable scenario. Cases B and C show moderate levels of both pollutants, with case C having slightly higher sulfur dioxide emissions. Overall, case A exhibits the best performance in terms of minimizing carbon dioxide, sulfur dioxide, and nitrogen oxides emissions, whereas the base case results in the highest environmental impact.

**Fig. 14:**
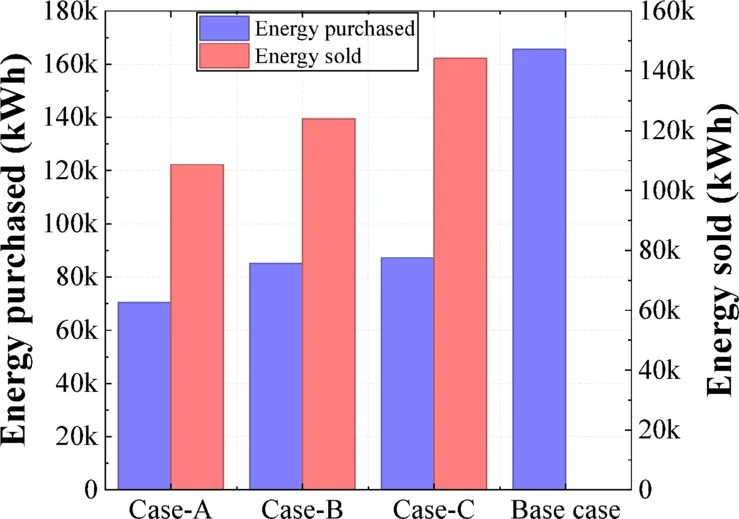
Comparison of energy transactions (purchase and sale) across different cases.

Figure 14 compares the amounts of energy purchased from and sold to the grid across different cases. The blue bars represent energy purchased (kWh), and the red bars indicate energy sold (kWh). Case-A shows a balanced approach with relatively equal energy purchased and sold. Case-B demonstrates a higher amount of energy purchased than sold, reflecting a slight imbalance in the system. Case-C shows the largest disparity between energy purchased and sold, with much higher energy purchased. The Base case exhibits the highest energy purchased and the least energy sold, indicating a poor overall energy balance. This figure highlights the efficiency and energy management of each case scenario.

### Optimum scenario

**Fig. 15:**
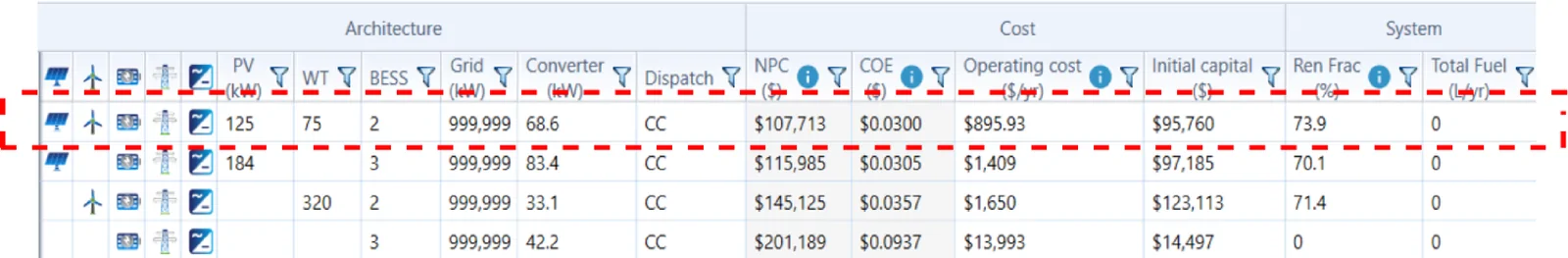
HOMER Pro simulation results comparing different microgrid architectures and their performance.

Based on the analysis, case A is identified as the most efficient configuration, offering an optimal balance between economic viability and environmental performance. This configuration incorporates a high share of RE, reducing dependence on fossil fuels. It is characterized by manageable capital and operating costs, providing a cost-effective solution without compromising system performance. Additionally, case A minimizes energy waste, such as excess electricity and unmet load, while ensuring a reliable energy supply. With its low environmental impact and focus on renewable sources, case-A stands out as the optimal choice for long-term sustainable performance and operational efficiency, making it the ideal choice among the alternatives. Figure 15 illustrates a comparison of multiple microgrid architectures, highlighting their performance across economic, environmental, and operational metrics.

**Fig. 16:**
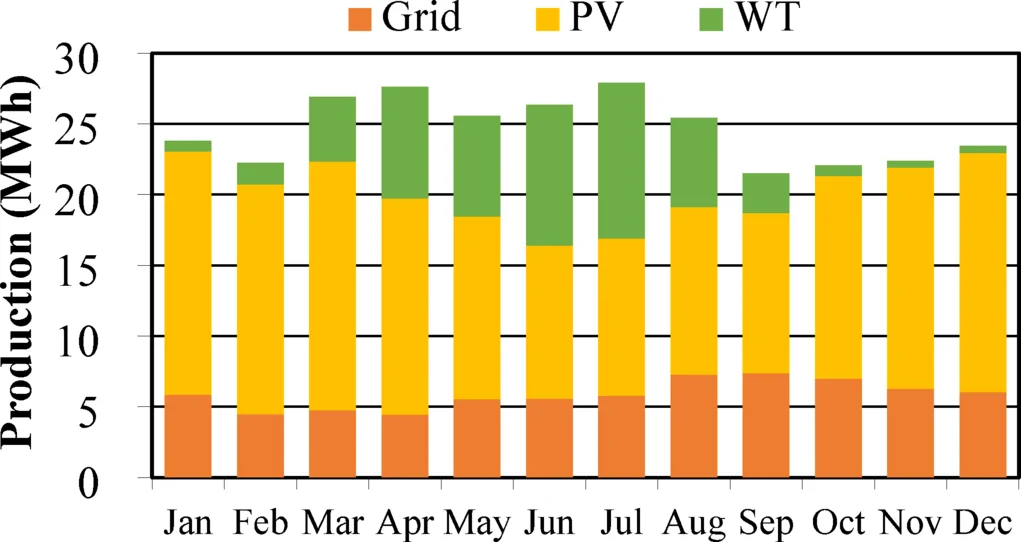
Monthly power output distribution across Solar PV, WT, and Grid for case A.

Figure 16 depicts the monthly energy output for the optimal scenario, illustrating the portion provided by PV, WT, and the grid. Solar PV generates 58.0% of the total energy, while WT contributes 18.1%, and the remaining 23.9% is purchased from the Grid. This energy mix ensures a high RF of 73.9%, indicating high dependence on RE sources throughout the year. The Solar PV system consistently performs well, while WT provides supplementary power. The utility grid acts as a backup source when renewable production is insufficient, ensuring a stable and reliable energy supply. This optimal configuration effectively balances renewable generation and grid usage for sustainability and cost efficiency.

**Fig. 17:**
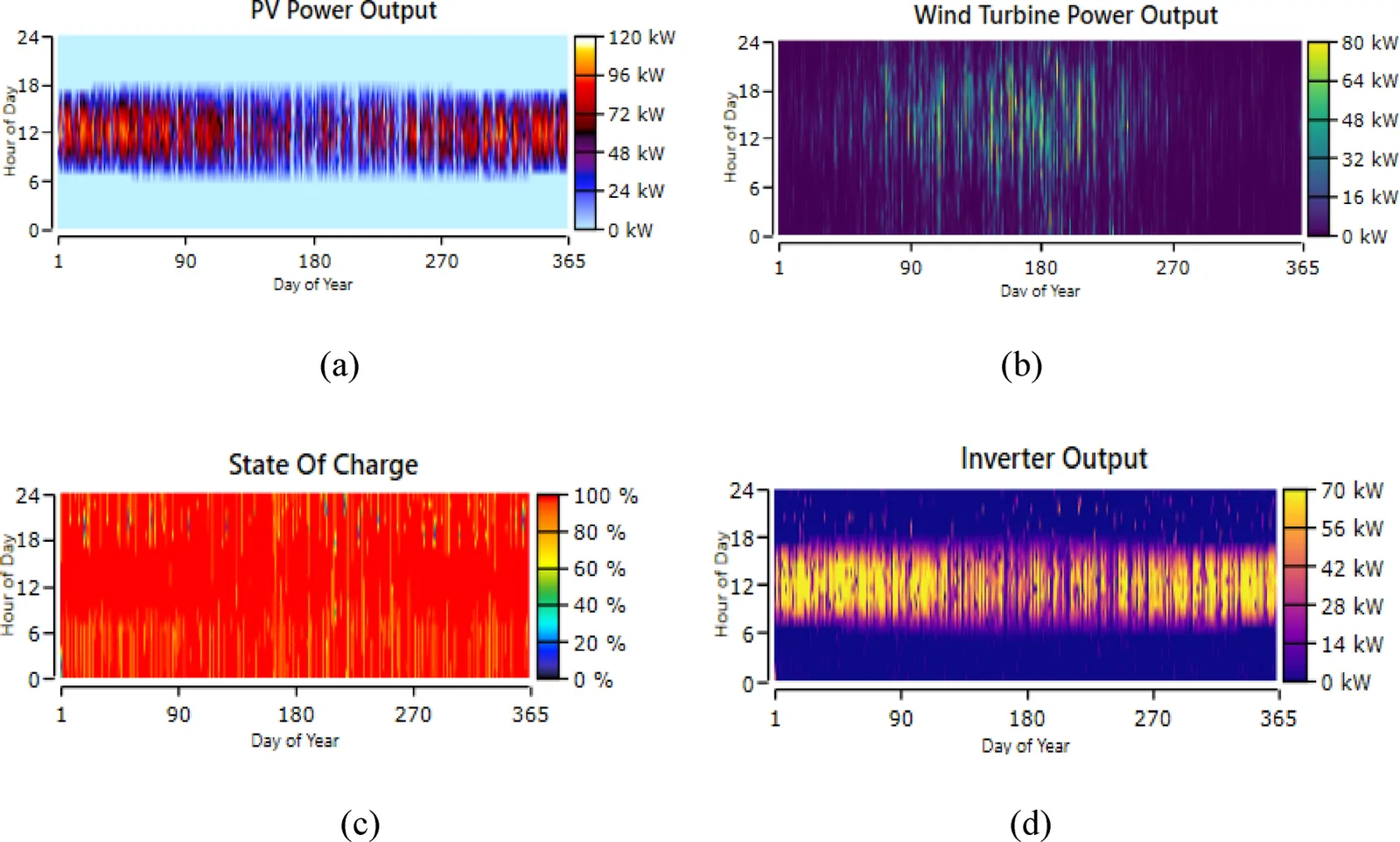
Yearly Energy Production and Storage Summary: (**a**) Solar PV Output (kW), (**b**) WT Output (kW), (**c**) Battery SOC (%), and (**d**) Inverter Output (kW).

Figure 17 provides a clear overview of how key RE systems performed throughout the year. Figure 17a shows the power output of the Solar PV system throughout the year, with the power output ranging from 0 to 120 kW. The x-axis denotes the days of the year, and the y-axis indicates the hours of the day, presenting how solar generation fluctuates daily and seasonally. Figure 17b depicts the wind turbine’s power output, which ranges from 0 to 80 kW. Similar to the PV output, it shows the daily and seasonal changes in wind power generation. Figure 17c illustrates the SOC of the BESS over the year. The SOC fluctuates between 0% and 100%, reflecting how energy is stored and utilized throughout the year. Figure 17d presents the inverter output, which converts stored energy to usable power, ranging from 0 to 70 kW. It captures the dynamics of energy conversion and delivery to the grid or load.

**Fig. 18:**
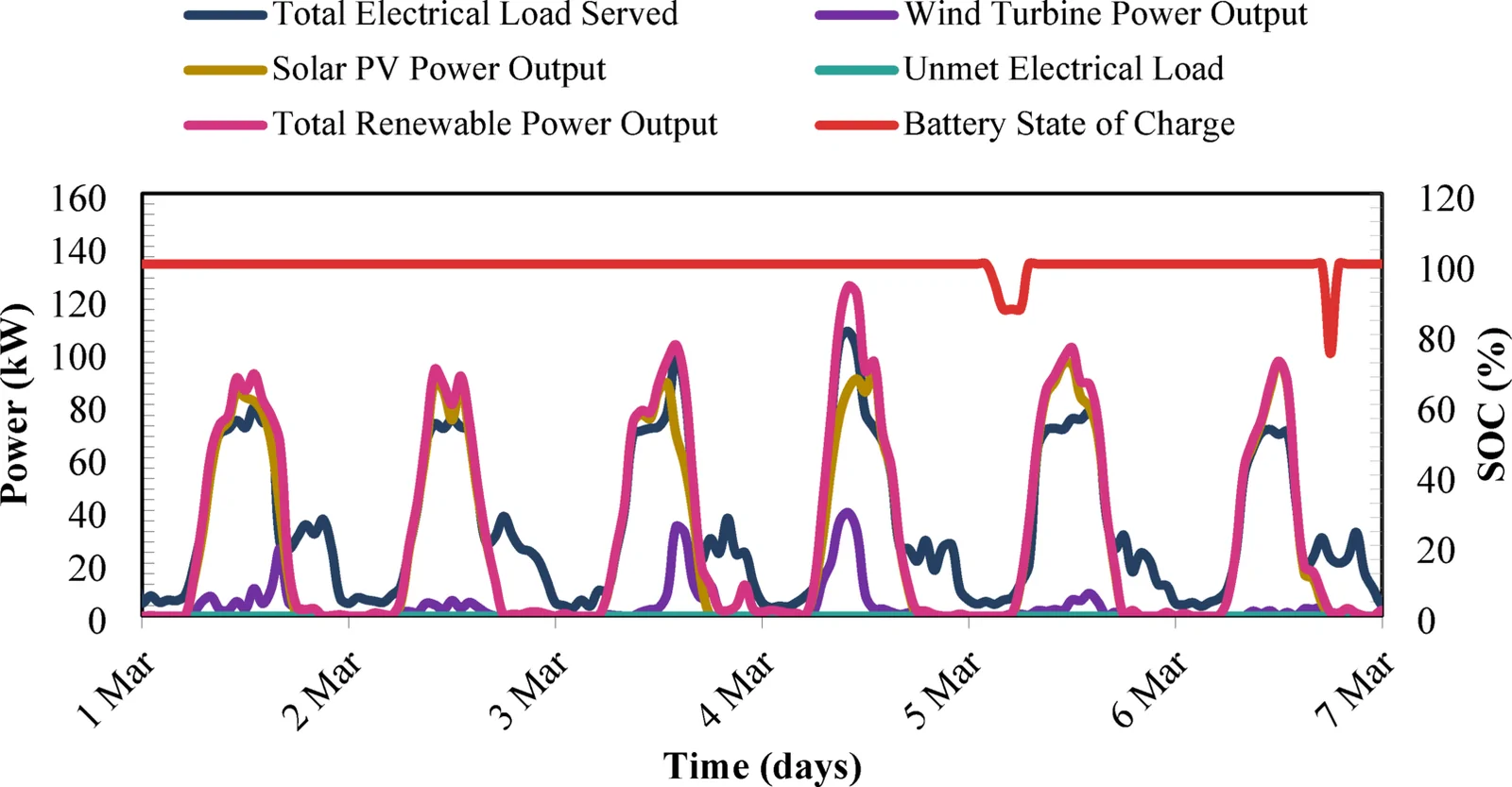
Hourly renewable generation, load served, unmet demand, and battery SOC.

Figure 18 presents the time series of RE generation, battery performance, and electrical load from March 1 to March 7. Solar PV output exhibits clear peaks around midday, driven by the availability of sunlight. WT power output complements this, contributing to total renewable energy generation, which fluctuates throughout the day in response to both solar and wind conditions. The battery maintains a near-full state of charge, ensuring a stable energy supply even when renewable generation decreases. As a result, the total electrical load is consistently met, with only minor periods of unmet demand, which are quickly compensated for by the battery. This highlights the efficient integration of solar, wind, and storage systems to manage variable power production and maintain a reliable energy supply. The figure demonstrates the robustness of the system in balancing RE sources with storage and system load demands across different times of the day.

**Fig. 19:**
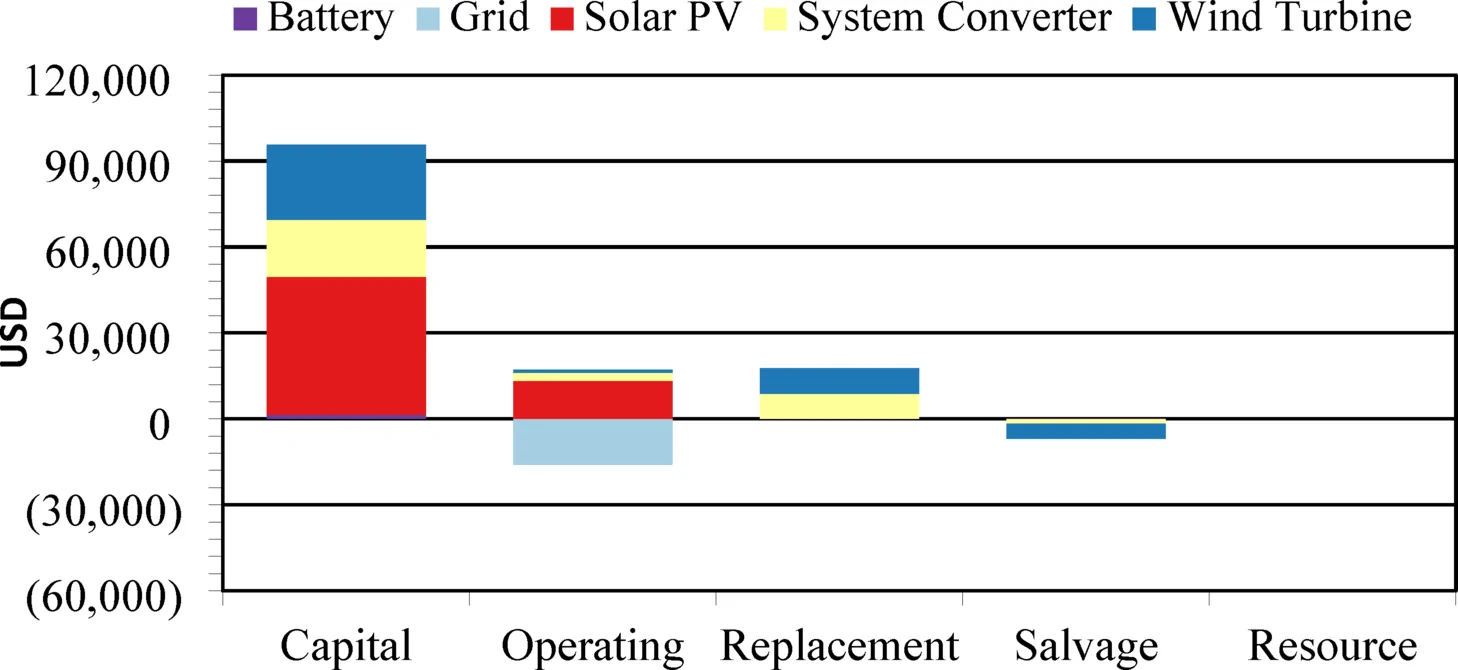
Cost breakdown for system components.

Figure 19 provides a detailed breakdown of the NPC for various system components, categorized into capital, operating, replacement, and residual costs. Solar PV incurs the highest capital expense, totaling $48,107, and also contributes significantly to operating costs at $13,337. The grid, on the other hand, shows a notable negative O&M of -$16,006, reflecting income or savings from grid-related operations. Replacement costs are primarily driven by the system converter and wind turbine, with respective costs of $8,845 and $8,902. In terms of salvage, the wind turbine leads with a reduction of -$5,095, indicating its contribution to lowering overall costs through salvage value. The breakdown reveals the financial structure of the system, emphasizing the significant costs associated with RE components such as solar PV, while showing the grid’s role in offsetting some operating expenses. The total NPC of the system is $107,713, which includes all these costs and savings.

**Fig. 20:**
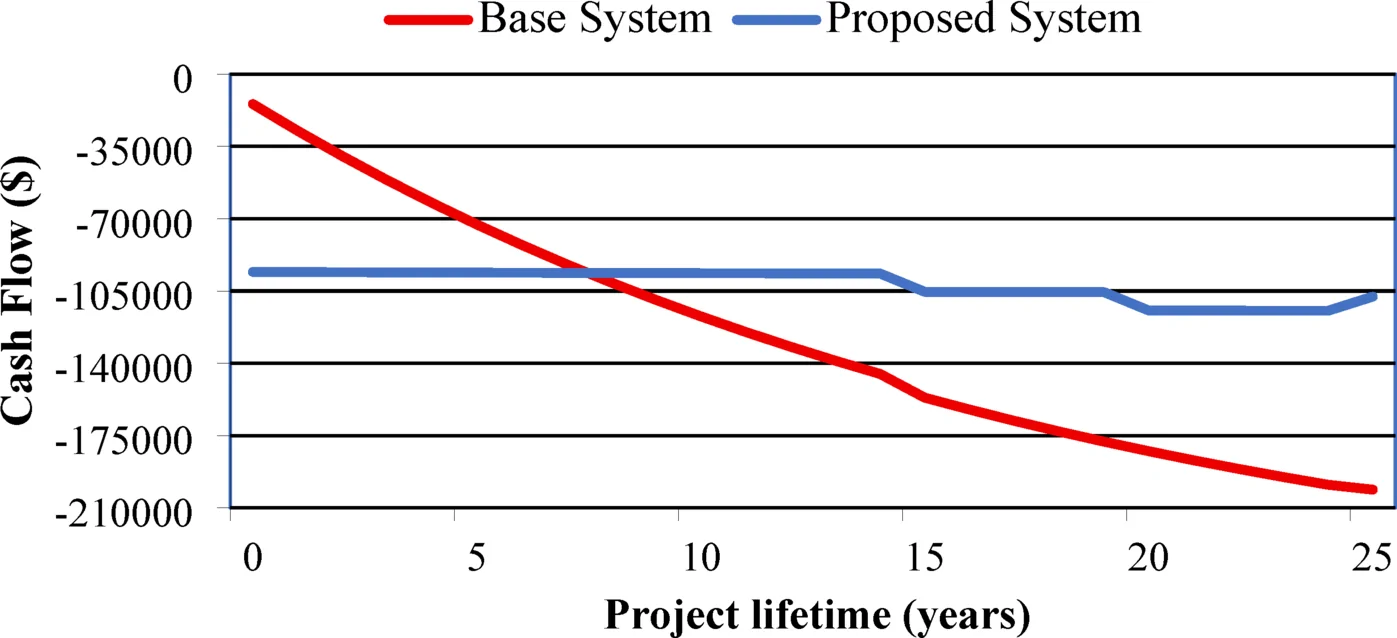
Lifetime cumulative cash flow.

Figure 20 shows the cash flow comparison between the existing and proposed system configurations, which demonstrates significant improvements in both economic and operational performance. The current system, which relies on 150 kWh of battery capacity, incurs annual operating costs of $13,993. In contrast, the proposed system, integrating 125 kW of PV and 75 kW of wind capacity, reduces these costs to $895.93 per year, highlighting substantial operational savings. Economically, the proposed system shows a NPV of $93,476, with an initial capital investment of $81,263. The system’s financial viability is further supported by a simple payback period of 5.98 years, ROI of 12.1%, and an IRR of 16%, making it an attractive investment both financially and environmentally.

### Sensitivity analysis results

Sensitivity analysis examines the impact of variations in input variables on model outputs, providing insights into which factors significantly influence the results. This method helps in identifying key parameters, quantifying their impact, and understanding the robustness of the model under varying conditions. Table 6 evaluates that this study applies a ± 20% variation to meteorological, economic, and system infrastructure parameters within HOMER Pro to assess their impact on RE system efficiency. Meteorological factors like solar radiation, wind speed, and temperature affect energy generation. Economic factors, including inflation rate, nominal discount rate, power price, and sellback rate, influence the financial feasibility of the system. Infrastructure parameters such as hub height, grid failure frequency, and repair times affect reliability and system stability. By conducting this analysis, HOMER Pro identifies the most sensitive variables, providing insights for optimizing system design, cost-effectiveness, and long-term sustainability under variable conditions.

**Table 6:** List of input sensitive variables with specified values.

Factor	Input Sensitive Variable	Values
Meteorological Data	Solar Radiation (kWh/m²/day)	2.75, 3.67, 4.58, 5.49, 6.40
	Wind Speed (m/s)	2.39, 3.19, 3.98, 4.78, 5.57
	Temperature (°C)	15.29,20.39,25.49,30.58,35.68
Economic Parameter	Inflation Rate (%)	4.8,6.4,8,9.6,11.2
	Nominal Discount Rate (%)	8.4,11.2,14,16.8,19.6
	Power Price ($/kWh)	0.048,0.064,0.08,0.096,0.112
	Sellback Rate ($/kWh)	0.024,0.032,0.04,0.048,0.056
Infrastructure and Reliability	Hub Height (m)	10.2,13.6,17,20.4,23.4
	Grid Failure Frequency (1/yr)	240,320,400,480,560
	Grid Mean Repair Time (h)	0.6,0.8,1,1.2,1.4
	Grid Variation Repair Time (%)	30, 40, 50, 60, 70

**Fig. 21:**
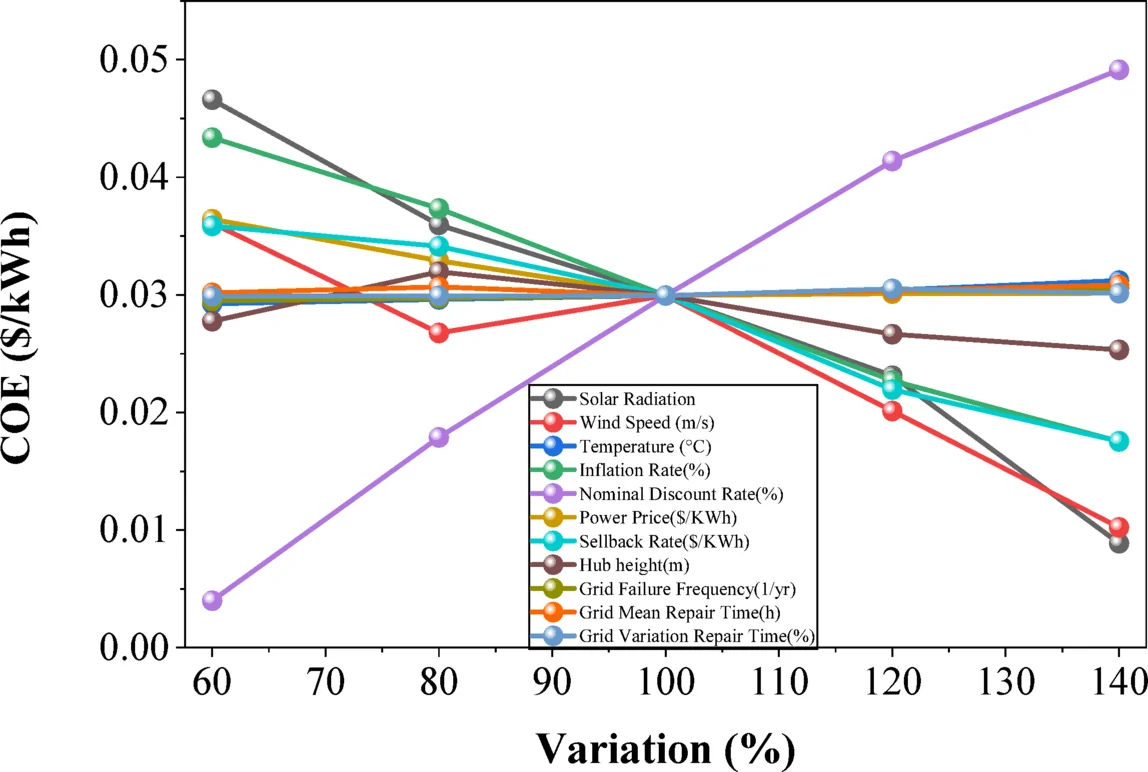
Impact of different parameters: Meteorological Data, Economic parameter, and Infrastructure and reliability on COE in RE systems.

The sensitivity analysis conducted in HOMER Pro evaluates how different parameters affect the COE relative to the baseline value of 100%. Figure 21 evaluates the sensitivity of COE to various parameters. Meteorological data showed considerable impacts: solar radiation caused a 107.4% increase at 60% variation, while at 140% it led to a 73.7% decrease. Wind speed caused a 15.3% increase at 60%, and a significant 89.4% decrease at 140%. Temperature showed smaller effects, decreasing by 30.4% at 60% and increasing by 35.1% at 140%. Economic parameters like inflation rate saw a 79.1% decrease at 140%, while nominal discount rate exhibited the greatest variability, decreasing by 95.5% at 60% and increasing by 113.6% at 140%. Power price caused a 0.3% increase at 140%, and the sellback rate decreased the COE by 24.4% at 140%.

Table 7 outlines the ranking of parameters based on sensitivity influencing the COE. The parameters are ranked based on the extent of their impact on COE, from those with the highest to the lowest influence. Nominal discount rate and solar radiation show significant variability in COE, making them highly sensitive factors. Wind Speed and Temperature also exhibit moderate effects, although their influence is less pronounced. On the other hand, hub height causes minimal changes in COE, positioning it as the least impactful parameter in the analysis.

**Table 7:** Ranking of parameters based on their sensitivity to COE.

Rank	Parameter	Approximate COE range, $/kWh	Reason
1	Nominal Discount Rate	0.004–0.049	Exhibits the largest variability in COE, indicating high sensitivity with significant fluctuations.
2	Solar Radiation	0.009–0.046	Has the greatest impact on COE, showing large increases and decreases depending on variations.
3	Inflation Rate	0.017–0.043	Strong negative correlation with COE, consistently decreasing COE as the inflation rate increases.
4	Wind Speed	0.010–0.035	Shows significant fluctuations in COE, indicating its high sensitivity to wind speed variations.
5	Temperature	0.025–0.030	Demonstrates moderate variability, affecting COE with both positive and negative changes.
6	Sellback Rate	0.030–0.036	Influences COE with notable decreases, highlighting its impact on cost efficiency.
7	Power Price	0.030–0.035	Shows a small yet consistent impact on COE, with minimal variations in response to price changes.
8	Hub Height	0.025–0.030	Displays moderate sensitivity, with changes in COE based on varying hub heights.
9	Grid Failure Frequency	0.028–0.031	Minimal impact on COE, with slight fluctuations observed across variations.
10	Grid Mean Repair Time	0.030–0.036	Has a lower impact on COE, with minimal changes observed.
11	Grid Variation Repair Time	0.029–0.030	Shows the least sensitivity, with negligible changes in COE despite variations.

**Fig. 22:**
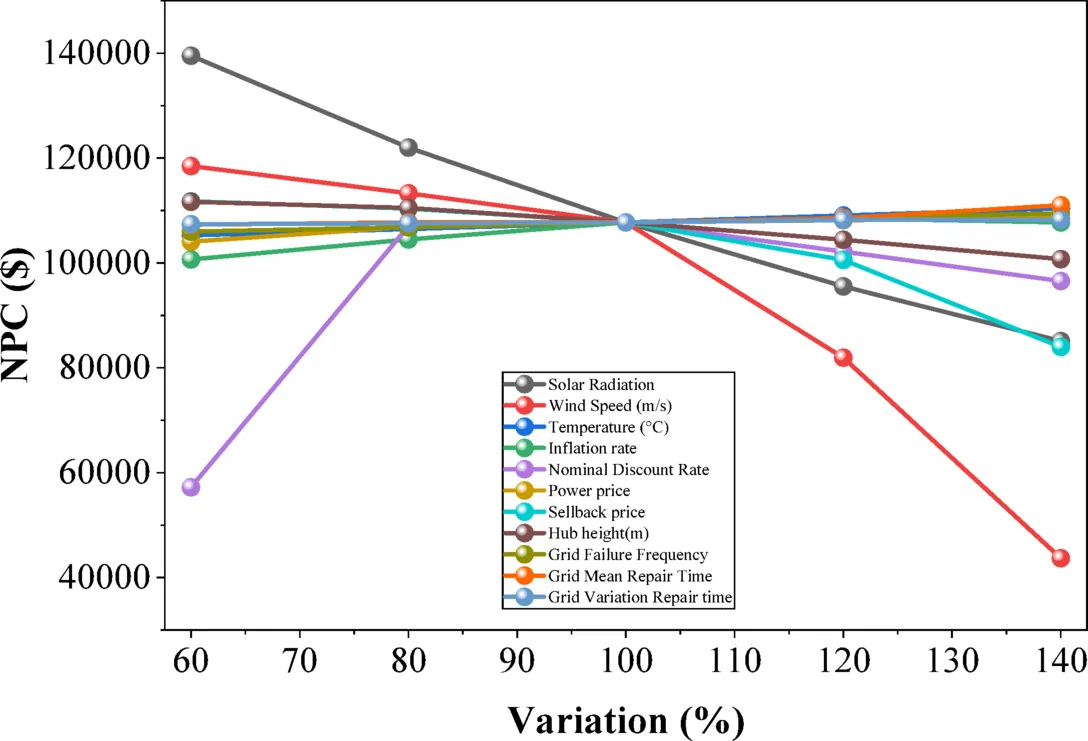
Impact of different parameters: Meteorological Data, Economic parameter, and Infrastructure and reliability on NPC in RE systems.

Figure 22 evaluates the sensitivity of NPC to various parameters. Meteorological data showed significant variability: solar radiation had a marked decrease of 39.3% at 140% variation and 13.1% at 60%. Wind speed decreased by 63.1% at 140% and increased by 3.7% at 60%. Temperature showed a smaller effect, increasing by 4.9% at 140% and 1.1% at 60%. Economic parameters like inflation rate decreased NPC by 6.4% at 140%, while nominal discount rate exhibited large variability, decreasing by 10.7% at 140% and increasing by 87.4% at 60%. Power price and sellback rate showed slight impacts, with power price decreasing by 2.6% at 60% and sellback rate decreasing by 24.4% at 140%. Infrastructure parameters such as grid mean repair time, grid failure frequency, grid variation repair time, and hub height had smaller effects, with variations between − 10.8% and + 3.1%. These results highlight the varying sensitivity of NPC to meteorological and economic factors.

Table 8 ranks the parameters based on their sensitivity to NPC. It evaluates how different factors, such as Nominal Discount Rate, Solar Radiation, Wind Speed, and others, influence the NPC. Parameters like Nominal Discount Rate and Solar Radiation exhibit the largest fluctuations in NPC, which places them at the top of the ranking, indicating high sensitivity. Conversely, Grid Failure Frequency and Grid Variation Repair Time show minimal variations in NPC, placing them at the bottom of the list. The table emphasizes the significance of each parameter in determining the cost-effectiveness of the system.

**Table 8:** Ranking of parameters based on their sensitivity to NPC.

Rank	Parameter	Approximate NPC range, $	Reason
1	Wind Speed	43,000–118,000	Exhibits the highest variability in NPC, showing both significant increases and decreases across the range of variations.
2	Solar Radiation	84,000–139,000	Strong impact on NPC with substantial decreases at higher variations, reflecting the influence of energy generation potential.
3	Nominal Discount Rate	56,000–108,000	Moderate influence on NPC, with clear changes as the wind speed increases or decreases.
4	Grid Mean Repair Time	105,000–111,000	Has a consistent effect on NPC, with slight increases at higher values, but a steady positive trend.
5	Power Price	104,000–108,000	Displays a smaller range of impact on NPC, but still shows noticeable changes as temperature shifts.
6	Sellback Rate	100,000–108,000	Shows moderate sensitivity, but the variations in NPC are less pronounced compared to the other parameters.
7	Inflation Rate	100,000–110,000	Influences NPC, but with a less significant variation compared to most of the other economic factors.
8	Temperature	106,000–108,000	Demonstrates minimal sensitivity, with small fluctuations observed in NPC, indicating its relatively low impact on system costs.
9	Grid Failure Frequency	106,000–109,000	Minimal impact on NPC, showing small changes across variations, suggesting it is less influential compared to other factors.
10	Hub Height	107,000–108,000	Exhibits the lowest variation in NPC, indicating minimal sensitivity to changes in grid repair times.
11	Grid Variation Repair Time	≈ 108,000	Shows the least sensitivity, with very small changes in NPC despite variations, marking it as the least impactful parameter.

**Fig. 23:**
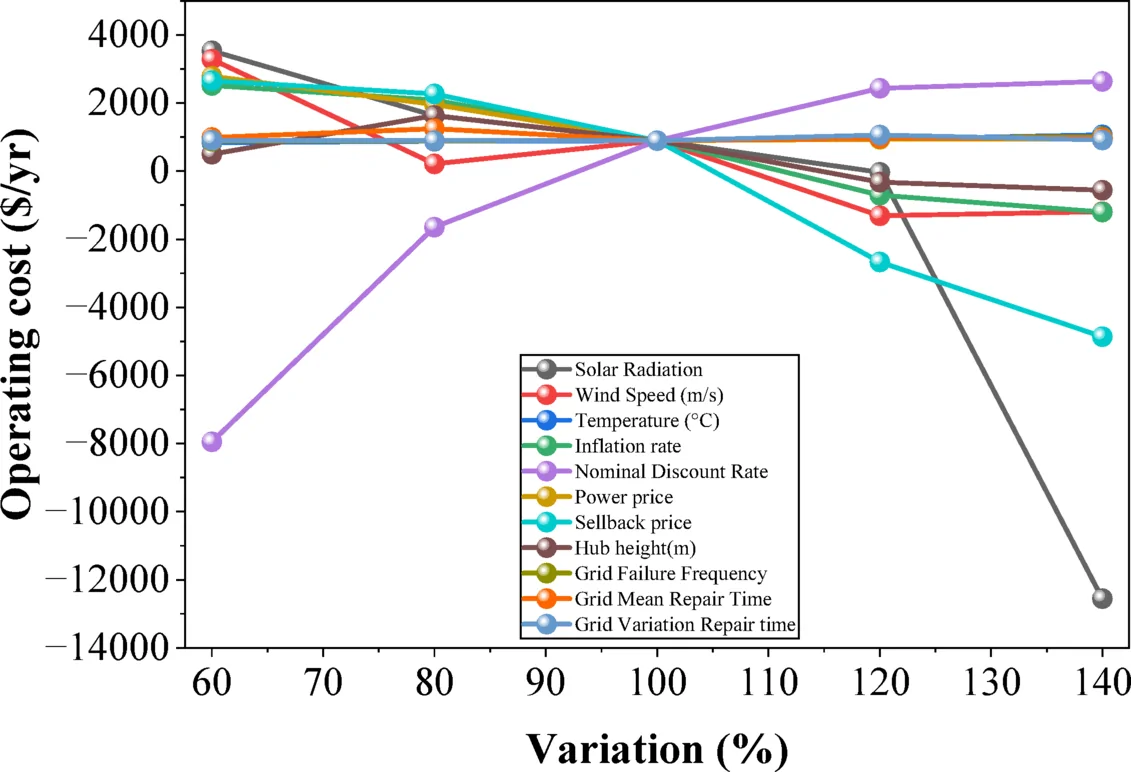
Impact of different parameters: Meteorological Data, Economic parameter, and Infrastructure and reliability on Operating cost in RE systems.

Figure 23 shows how operating costs vary in response to various parameters and their variations. The solar radiation parameter showed significant variability, with a drastic increase in operating costs at 60% variation (3534.66 $/yr) and a sharp decrease at 140% variation (-12552.63 $/yr). Wind speed exhibited a noticeable decrease in operating cost at 80% variation (214.40 $/yr) but escalated at higher values, showing substantial negative costs at 140%. Temperature exhibited a more consistent increase, rising from 837.09 $/yr at 60% to 1063.97 $/yr at 140%, indicating a moderate but steady rise. In terms of economic parameters, nominal discount rate displayed significant fluctuations, with a large negative impact at 60% (-7947.07 $/yr) and a positive effect at 140% (2637.27 $/yr). Power price and sellback price had relatively more moderate effects, although the sellback price at 140% saw a large negative value (-4859.41 $/yr).

Table 9 ranks the parameters based on their sensitivity to operating costs. Parameters like solar radiation and nominal discount rate exhibited the greatest fluctuations, making them the most influential in determining operating costs. Wind speed and sellback price also had substantial effects. Power price and temperature showed moderate sensitivity, affecting the operating costs in a consistent manner. Hub height, inflation rate, and grid-related factors had a more limited impact, with relatively smaller variations in operating costs observed. This ranking helps highlight the most critical parameters influencing operational expenses in the system.

**Table 9:** Ranking of parameters based on their sensitivity to Operating cost.

Rank	Parameter	Approximate operating-cost range, $/yr	Reason
1	Solar Radiation	−12,700 to 3,400	Exhibits the highest sensitivity, showing dramatic fluctuations in operating cost, from large increases to significant decreases.
2	Nominal Discount Rate	−8,000 to 2,500	Shows the second largest variation in operating cost, with large negative values at lower variations and positive shifts at higher levels.
3	Wind Speed	−5,000 to 2,500	Demonstrates high sensitivity, with significant changes in operating cost, especially at higher variations.
4	Power Price	−1,400 to 3,200	Impacts operating cost moderately, with fluctuations at both lower and higher variations.
5	Sellback Price	−1,300 to 2,400	Exhibits noticeable negative impact at higher variations, making it an important factor in operating cost sensitivity.
6	Temperature	−700 to 1,000	Shows moderate sensitivity, with a steady increase in operating cost as temperature rises.
7	Hub Height	800 to 1,200	Displays a moderate but consistent impact on operating cost, with a decrease at higher variations.
8	Inflation Rate	700 to 900	Has a consistent but smaller effect on operating cost, with a steady decrease as inflation increases.
9	Grid Failure Frequency	900 to 3,100	Demonstrates a mild impact on operating cost, with small fluctuations across the variations.
10	Grid Mean Repair Time	−700 to 900	Shows relatively minimal variation in operating cost, with only slight changes across different variations.
11	Grid Variation Repair Time	600 to 900	Displays the least sensitivity, with very small fluctuations in operating cost despite variations.

**Fig. 24:**
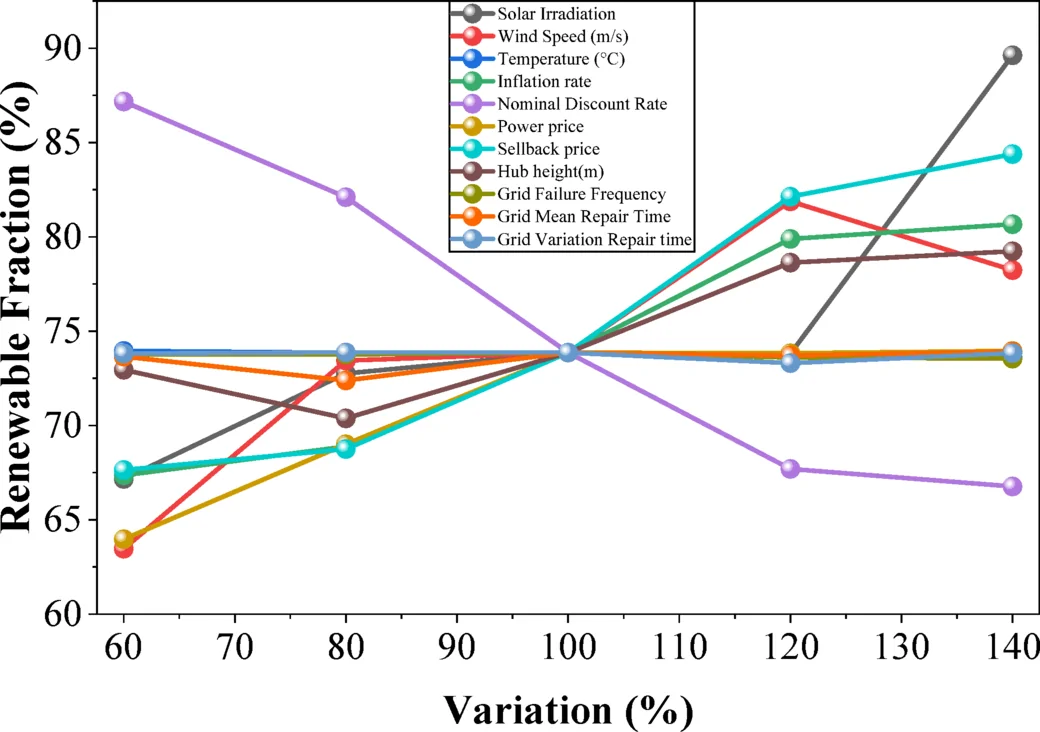
Impact of different parameters: Meteorological Data, Economic parameter, and Infrastructure and reliability on RF in RE systems.

Figure 24 presents the sensitivity analysis of the RF in relation to various parameters and their variations. Solar radiation exhibited a notable increase in RF, from 67.17% at 60% variation to 89.62% at 140%, indicating a strong dependency on solar resource availability. Wind speed displayed a more modest influence, with variations from 63.47% at 60% to 78.24% at 140%. Temperature had the least effect on renewable fraction, remaining relatively stable at 73.87% at 100% and showing a slight decrease at higher variations, reaching 73.65% at 140%. Among the economic parameters, the nominal discount rate caused the greatest fluctuation, dropping from 87.18% at 60% to 66.77% at 140%. Power price and sellback price displayed moderate changes, with power price maintaining a steady renewable fraction around 73.87%, while the sellback price increased from 67.65% at 60% to 84.38% at 140%. Infrastructure parameters like hub height showed a moderate increase, from 72.96% at 60% to 79.24% at 140%, reflecting its importance in renewable energy contribution.

Table 10 ranks parameters based on their sensitivity to RF. Solar radiation has the greatest effect, leading to substantial increases in RF as solar availability improves. Nominal discount rate and sellback price also exhibit significant fluctuations, highlighting their importance in renewable energy contribution. Wind speed and hub height display moderate sensitivity, with wind speed contributing notably to the RF. In contrast, temperature and grid-related factors show minimal impact on RF, indicating lower sensitivity relative to other parameters.

**Table 10:** Ranking of parameters according to their sensitivity to RF.

Rank	Parameter	Approximate RF range, %	Reason
1	Solar Radiation	67.2–89.6	Exhibits the largest impact on the renewable fraction, with a significant increase at higher variations.
2	Nominal Discount Rate	66.8–87.2	Shows the most significant fluctuation, decreasing sharply at higher variations.
3	Sellback Price	64.0–84.3	Has a strong positive correlation, with a notable increase in RF as the sellback price rises.
4	Wind Speed	63.5–79.9	Demonstrates a noticeable increase in renewable fraction, though not as significant as solar radiation.
5	Hub Height	67.5–84.3	Displays a moderate effect, with RF increasing gradually with higher hub height.
6	Power Price	70.2–79.2	Exhibits minor changes in RF but remains relatively stable across variations.
7	Temperature	72.8–73.9	Shows minimal impact on RF, with only small variations observed across different temperature levels.
8	Grid Failure Frequency	73.5–73.9	Has a limited effect, causing slight changes in RF.
9	Grid Mean Repair Time	63.8–73.9	Displays a small impact on RF, with minimal changes observed.
10	Grid Variation Repair Time	73.5–73.9	Shows the least sensitivity, with only minor changes in RF despite variations.

Table 11 presents the variations in NPC and COE in response to changes in the capital, replacement, and O&M costs of key components used in a microgrid system. These components include PV, WT, BESS, and converter. The table shows how changes in the cost of each component, whether a 20% increase or decrease, impact both NPC and COE, offering valuable insights into their economic effects. These variations are crucial for understanding the sensitivity of system costs to changes in component pricing, which is essential for optimizing cost-efficiency in microgrid systems.

**Table 11:** Effect of 20% variations in component costs on NPC and COE.

Components	Changes of capital, replacement, and O&M cost	Variation of NPC (%)	Variation of COE (%)
PV	20% (decrease)	−14.47%	−25.33%
	20% (increase)	9.69%	16.67%
WT	20% (decrease)	−11.79%	−29.99%
	20% (increase)	3.42%	−3.66%
BESS	20% (decrease)	−0.27%	−0.33%
	20% (increase)	0.27%	0.0%
Converter	20% (decrease)	−5.92%	−14.67%
	20% (increase)	4.99%	8.67%.

**Fig. 25:**
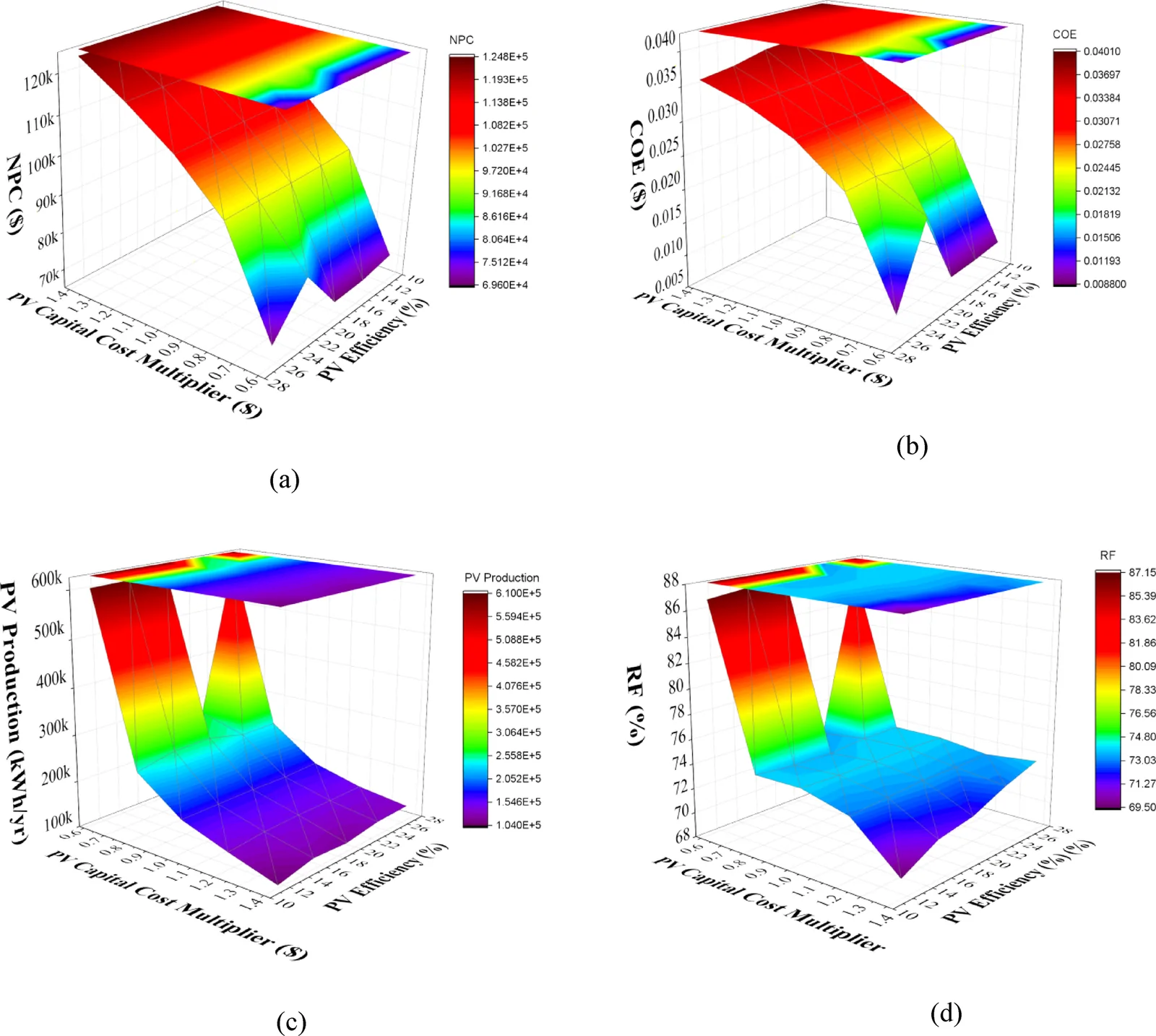
Sensitivity of PV capital cost multiplier and PV efficiency on system performance: (**a**) NPC, (**b**) COE, (**c**) PV production, and (**d**) RF.

Figure 25 demonstrates analysis of how PV system performance responds to changes in PV capital expenditure multiplier and efficiency. Figure 25a shows the NPC, where increasing the capital cost multiplier or decreasing efficiency results in a higher NPC. Figure 25b illustrates the COE, which similarly increases with higher capital costs and lower efficiencies. Figure 25c presents the annual PV production, indicating that increasing system efficiency leads to a noticeable rise in energy production, while higher capital costs reduce it. Figure 25d displays the RF, which decreases as the capital cost multiplier rises or efficiency declines. The analysis reveals that the system’s economic performance, reflected in NPC and COE, is most sensitive to changes in capital cost, whereas PV production and RF are more influenced by efficiency. These findings emphasize the key role of optimizing both capital investment and efficiency to improve the economic efficiency and environmental sustainability of PV systems.

**Fig. 26:**
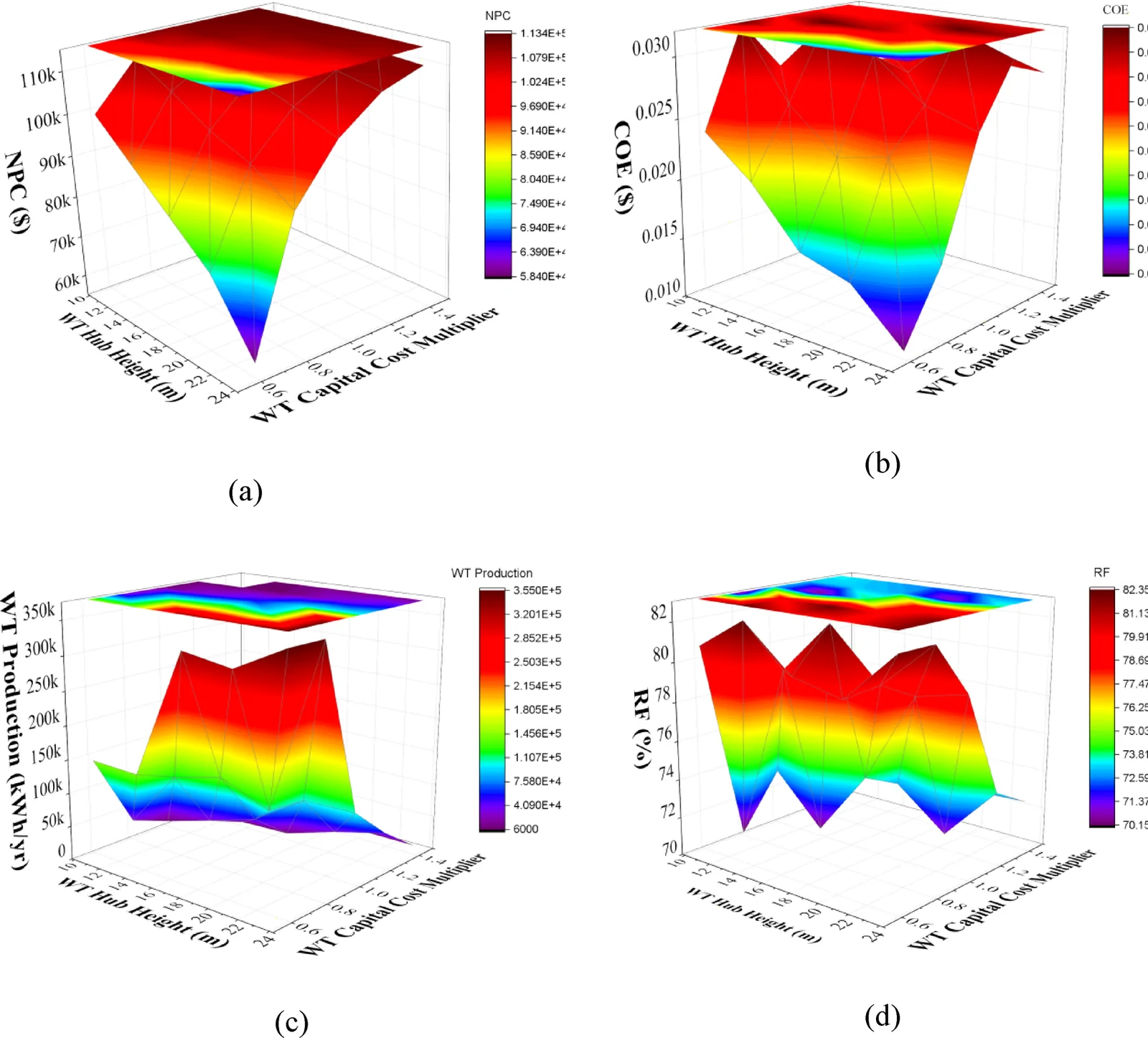
Sensitivity of WT hub height and WT capital cost multiplier on system performance: (**a**) NPC, (**b**) COE, (**c**) PV production, and (**d**) RF.

Figure 26 illustrates how the performance of the system fluctuates with changes in the hub height and WT capital cost multiplier. In Fig. 26a, the NPC increases as the capital cost multiplier rises. However, higher hub heights help reduce the NPC, suggesting that optimal hub height is crucial for cost control. Figure 26b shows COE, which similarly increases with higher capital costs, while higher hub heights lower the COE, indicating that increased hub height improves energy cost efficiency. Figure 26c demonstrates that WT energy production primarily depends on hub height, with higher hub heights leading to significantly higher energy output. In contrast, the impact of the capital cost multiplier on production is relatively smaller. Figure 26d presents the RF, which increases with higher hub heights and decreases with higher capital cost multipliers. This analysis emphasizes that optimizing hub height and managing capital costs are essential for enhancing both the economic viability and RE contribution of WT systems.

### Performance correlation of microgrid components

**Fig. 27:**
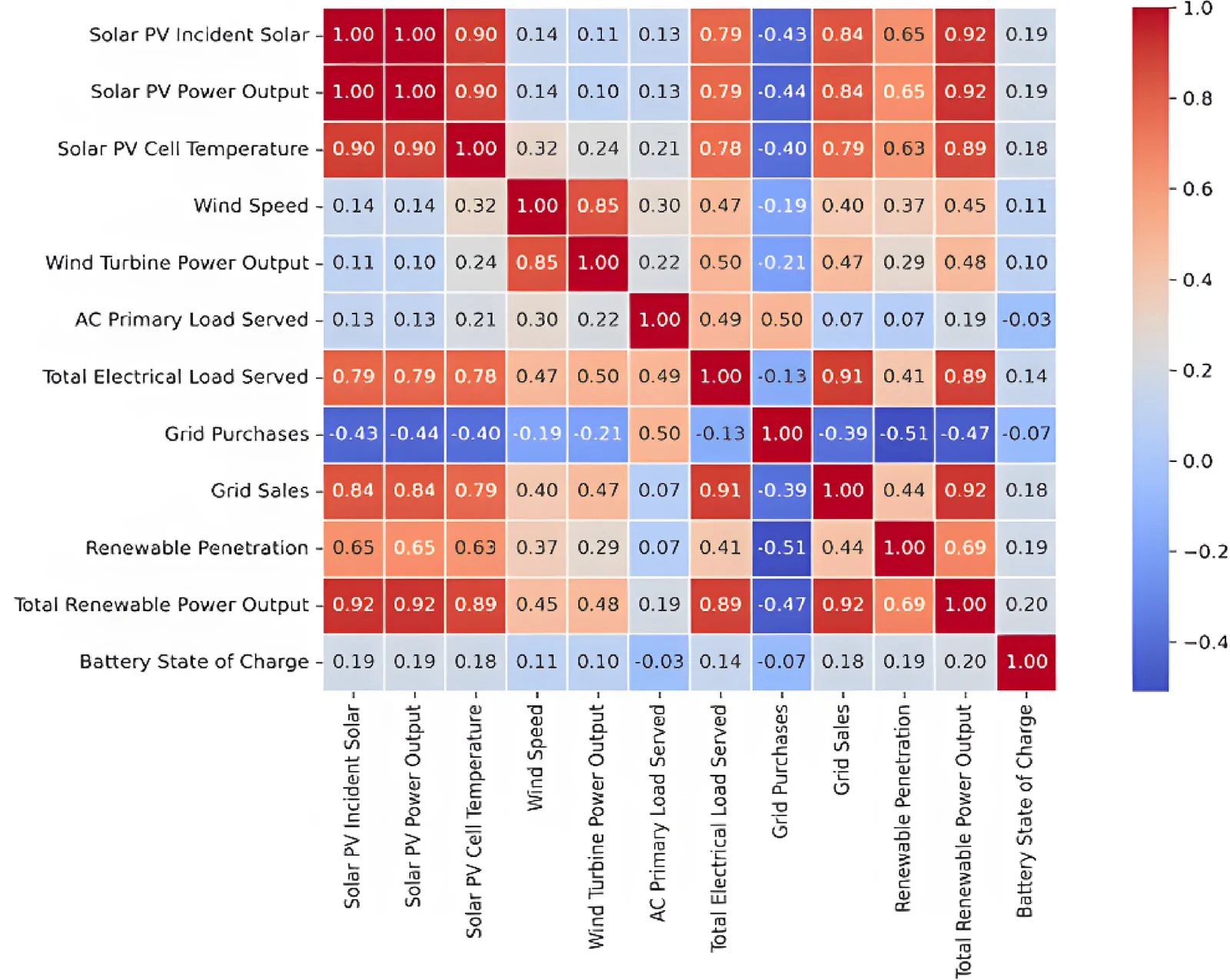
Correlation matrix of critical variables in solar, wind, and grid energy systems.

The correlation matrix in Fig. 27 explores the relationships between key variables influencing the performance and operation of RE systems, focusing on solar and wind energy as well as grid interactions. It includes variables such as PV incident solar, PV power output, PV cell temperature, wind Speed, and WT power output, crucial for assessing energy production efficiency. Additionally, grid-related metrics, including AC primary load served, total electrical load served, grid imports, and grid exports, provide insights into energy consumption and distribution. The matrix highlights the strength and direction of correlations, shedding light on the interdependencies that affect system performance. The following visualization offers a clear representation of these relationships, enhancing the understanding of renewable energy output and grid stability.

### Comparison with previously published works

To benchmark the proposed HRES against existing studies, a comparative analysis with recently published works is presented in Table 12. The comparison considers key techno-economic and environmental indicators, including system configuration COE, NPC, RF, and CO₂ emission reduction, providing a clear context for assessing the performance and competitiveness of the recommended system.

**Table 12:** Comparison of the proposed system with previously published studies.

System Structure	Location	System type & Category	RF (%)	NPC ($) and COE ($/kWh)
PV-WT-DG-BESS^75^	Kutubdia Island, Bangladesh	Off-grid Community	74%	$17.6 M $0.236/kWh
PV-WT-DG-BESS-Grid^76^	Central Tilba, New South Wales, Australia	On-grid Community	---	$4.83 M $0.208/kWh
PV-DG-BESS^77^	Punjab, India	Off-grid Community	99.1	$2.34 M $28.93/kWh
PV-BESS-Grid^78^	Egypt	Off-grid Community	72	$58,000 $0.089/kWh
PV-Biogen-Grid^79^	Pabna, Bangladesh	On-grid University	64.2	$4,712,218 $0.0347/kWh
PV-WT-BESS-Grid^80^	Gujarat, India	On-grid Community	63.4	$1.48 M $0.087/kWh
PV-WT-Hydro-BESS^81^	Arandun, Nigeria	Off-grid Village	100	$55.7 M $0.26
PV-Grid^82^	Uttarakhan, Dhaka	On-grid Residential Complex	54.5	$70,448 $0.026
PV-WT-BESS-Grid^83^	El Aromo, Ecuador	On-grid Residential	---	$4,232,505 $0.09
PV-HT-EL-FC^84^	Oman	Off-Grid Community	100	$317,401,965 $0.97
PV-WT-PHS-Grid^41^	Tazarine, Morocco	On-grid Village	---	$262,596 $0.03831
PV-DG-BESS^85^	Malaysia	Off-grid Community	62.25	743,670 0.33
PV-DG-BESS^86^	Gyeonggi Province, South Korea	Off-grid Educational Institution	76	1,615,112 0.673
Grid-PV-BESS^87^	Heraklion, Crete, Greece	On-grid smart campus	60	738,306.47 ---
PV-WT-Grid^88^	Chitradurga District, Karnataka	On-Grid Community	37.8	633,352 0.109
The Proposed Work	Mithamain, Kishoreganj Bangladesh	On-grid Community	73.9%	$107,712.60 $0.02995/kWh

The comparative results demonstrate that the proposed PV-WT-battery-grid microgrid achieves a notably lower COE and NPC compared to many existing HRES reported in the literature. The high RF and substantial reduction in CO₂ emissions further underscore the environmental advantages of the proposed design. While some studies report higher REP or fully off-grid solutions, these often come at the expense of increased capital investment or operational costs. In contrast, the grid-connected hybrid configuration presented in this study offers a balanced and scalable solution, ensuring energy reliability during flood-induced grid outages while maintaining economic viability. These findings confirm the suitability of the suggested microgrid as a climate-resilient and cost-effective rural electrification solution for flood-prone wetland regions.

## Conclusion

This research highlights the potential of hybrid RE microgrids as a practical and sustainable solution for providing electricity to rural communities in flood-prone areas of Bangladesh, such as Mithamain Upazila. These communities often face unreliable power sources due to frequent flooding, which disrupts daily life and economic activities. By combining PV panels, wind turbines (WT), a battery energy storage system (BESS), and a power converter, the proposed system guarantees a stable electricity supply, even during seasonal flooding, offering a dependable energy solution when it’s most needed. The techno-economic analysis shows that the system offers a competitive cost of electricity (COE) of $0.02995/kWh, which is comparable to the cost of conventional rural electricity, making it an affordable option for these communities. Additionally, with a yearly CO2 reduction of 43,675 kg, the system contributes significantly to environmental sustainability by reducing harmful emissions. This environmental benefit aligns with global efforts to combat climate change and promotes cleaner energy alternatives for communities with limited access to electricity. The sensitivity analysis further demonstrates the system’s resilience, making it a reliable option under varying climatic and economic conditions. This resilience is vital for ensuring energy access, especially in regions that experience unpredictable weather patterns and economic challenges. This research emphasizes that hybrid microgrids are not only a feasible solution for rural electrification but also a practical and scalable approach to addressing energy challenges in other developing regions. By minimizing fossil fuel use and reducing ecological footprints, hybrid microgrids offer a promising path to sustainable energy access, climate change mitigation, and improved quality of life for vulnerable communities. These solutions can provide long-term energy security while helping to build a more sustainable future for the next generation.

## Data Availability

Data will be made available upon reasonable request from the corresponding author.

## Abbreviations

AC: Alternating current
BESS: Battery energy storage system
COE: Cost of energy
DG: Diesel generator
GEF: Grid emission factor
GHG: Greenhouse gas
GHI: Global horizontal irradiance
HOMER: Hybrid optimization of multiple energy resources
HRES: Hybrid renewable energy system
IRR: Internal rate of return
LCOE: Levelized cost of energy
NASA: National aeronautics and space administration
NPC: Net present cost
NPV: Net present value
NREL: National renewable energy laboratory
O&M: Operation and maintenance
PV: Photovoltaic
REP: Renewable energy penetration
RF: Renewable fraction
ROI: Return on investment
SAM: Stand-alone microgrid
SoC: State of charge
STC: Standard test conditions
WT: Wind turbine
